# The Impact of Monitoring HIV Patients Prior to Treatment in Resource-Poor Settings: Insights from Mathematical Modelling

**DOI:** 10.1371/journal.pmed.0050053

**Published:** 2008-03-11

**Authors:** Timothy B Hallett, Simon Gregson, Sabada Dube, Geoff P Garnett

**Affiliations:** 1 Department of Infectious Disease Epidemiology, Imperial College London, United Kingdom; 2 Biomedical Research and Training Institute, Harare, Zimbabwe; Harvard School of Public Health, United States of America

## Abstract

**Background:**

The roll-out of antiretroviral treatment (ART) in developing countries concentrates on finding patients currently in need, but over time many HIV-infected individuals will be identified who will require treatment in the future. We investigated the potential influence of alternative patient management and ART initiation strategies on the impact of ART programmes in sub-Saharan Africa.

**Methods and Findings:**

We developed a stochastic mathematical model representing disease progression, diagnosis, clinical monitoring, and survival in a cohort of 1,000 hypothetical HIV-infected individuals in Africa. If individuals primarily enter ART programmes when symptomatic, the model predicts that only 25% will start treatment and, on average, 6 life-years will be saved per person treated. If individuals are recruited to programmes while still healthy and are frequently monitored, and CD4^+^ cell counts are used to help decide when to initiate ART, three times as many are expected to be treated, and average life-years saved among those treated increases to 15. The impact of programmes can be improved further by performing a second CD4^+^ cell count when the initial value is close to the threshold for starting treatment, maintaining high patient follow-up rates, and prioritising monitoring the oldest (≥ 35 y) and most immune-suppressed patients (CD4^+^ cell count ≤ 350). Initiating ART at higher CD4^+^ cell counts than WHO recommends leads to more life-years saved, but disproportionately more years spent on ART.

**Conclusions:**

The overall impact of ART programmes will be limited if rates of diagnosis are low and individuals enter care too late. Frequently monitoring individuals at all stages of HIV infection and using CD4 cell count information to determine when to start treatment can maximise the impact of ART.

## Abstract 2

Using a stochastic model based on data from Africa, Timothy Hallett and colleagues find that starting HIV treatment based on regular CD4 monitoring, rather than on symptoms, would substantially increase survival.

## Editors' Summary

**Background.:**

Acquired immunodeficiency syndrome (AIDS) has killed more than 25 million people since the first case in 1981, and about 33 million people are currently infected with the human immunodeficiency virus (HIV), which causes AIDS. HIV destroys immune system cells (including CD4 cells, a type of lymphocyte), leaving infected individuals susceptible to other infections. Early in the AIDS epidemic, most HIV-positive individuals died within 10 years but in 1996, combination antiretroviral therapy (ART)—a mixture of powerful but expensive antiretroviral drugs—was developed. For HIV-positive people living in affluent, developed countries who could afford ART, AIDS then became a chronic disease, but for those living in low- and middle-income countries it remained a death sentence—ART was too expensive. In 2003, this lack of access to ART was declared a global health emergency and governments, international organizations, and funding bodies began to implement plans to increase ART coverage in developing countries.

**Why Was This Study Done?:**

The roll-out of ART in developing countries has concentrated so far on finding HIV-positive people who currently need treatment. In developing countries, these are often individuals who have AIDS-related symptoms such as recurrent severe bacterial infections. But healthy people are also being diagnosed as HIV positive during voluntary testing and at antenatal clinics. How should these HIV-positive but symptom-free individuals be managed? Should regular health-monitoring appointments be scheduled for them and when should ART be initiated? Management decisions like these will determine how well patients do when they eventually start ART, as well as the demand for ART and other health-care services. The full range of alternative patient management strategies cannot be tested in clinical trials—it would be unethical—but public-health officials need an idea of their relative effectiveness in order to use limited resources wisely. In this study, therefore, the researchers use mathematical modeling to investigate the impact of alternative patient management and ART initiation strategies on the impact of ART programs in resource-poor settings.

**What Did the Researchers Do and Find?:**

The researchers' mathematical model, which includes data on disease progression collected in Africa, simulates disease progression in a group (cohort) of 1,000 HIV-infected adults. It tracks these individuals from infection, through diagnosis and clinical monitoring, and into treatment and predicts how many will receive ART and their length of survival under different management scenarios and ART initiation rules. The model predicts that if HIV-positive individuals receive ART only when they have AIDS-related symptoms, only a quarter of them will ever start ART and the average life-years saved per person treated will be 6 years (that is, they will live 6 years longer than they would have done without treatment). If individuals are recruited to ART programs when they are healthy and are frequently monitored using CD4 cell counts to decide when to start ART, three-quarters of the cohort will be treated and 15 life-years will be saved per person treated. The impact of ART programs will be increased further, the model predicts, by preferentially monitoring people who are more than 35 years old and the most immunosuppressed individuals. Finally, strategies that measure CD4 cells frequently will save more life-years because ART is more likely to be started before the immune system is irreversibly damaged. Importantly for resource-poor settings, these strategies also save more life-years per year on ART.

**What Do These Findings Mean?:**

As with all mathematical models, the accuracy of these predictions depends on the assumptions built into the model and the reliability of the data fed into it. Also, this model does not estimate the costs of the various management options, something that will need to be done to ensure effective allocation of limited resources. Nevertheless, these findings provide several general clues about how ART programs should be implemented in poor countries to maximize their effects. Early diagnosis of infections, regular monitoring of patients, and using CD4 cell counts to decide when to initiate ART should all help to improve the number of life-years saved by ART. In other words, the researchers conclude, effectively managing individuals at all stages of HIV infection is essential to maximize the impact of ART.

**Additional Information.:**

Please access these Web sites via the online version of this summary at http://dx.doi.org/doi:10.1371/journal.pmed.0050053.

- Information from the US National Institute of Allergy and Infectious Diseases on HIV infection and AIDS.
- Information from the US Centers for Disease Control and Prevention on global HIV/AIDS topics (in English and Spanish)
- HIV InSite, comprehensive and up-to-date information on all aspects of HIV/AIDS from the University of California, San Francisco
- Information from Avert, an international AIDS charity, on HIV and AIDS in Africa and on HIV/AIDS treatment and care, including universal access to ART
- Progress toward universal access to HIV/AIDS treatment, the latest report from the World Health Organization (available in several languages)
- Guidelines for antiretroviral therapy in adults and adolescents are provided by the World Health Organization and by the US Department of Health and Human Services

## Introduction

For a decade, HIV-infected persons in developed countries whose clinical course is advancing towards AIDS have been offered combination antiretroviral therapy (ART); this practice has generated a sharp decline in the rate of AIDS deaths [1]. More recently, the humanitarian crisis associated with the general spread of HIV in many African countries has led to an unprecedented financial and logistical commitment to providing ART to those in need [2–4]. Activities now focus on identifying those currently requiring treatment rather than on how to monitor those with future treatment needs [5]. However, decisions need to be made about how to care for all HIV-infected individuals. These decisions will determine the prognosis when an individual initiates ART, and the future demand for ART and other health and social services across the population.

Survival of HIV-infected individuals on ART depends on the state of their immune system when treatment is begun [6,7]. Whilst in Western countries the decision to initiate ART is informed by a range of high-technology tools [8,9], in poorer countries a more pragmatic public-health approach has been adopted [10]. The World Health Organisation (WHO) has recommended using CD4^+^ cell counts to decide when treatment should be initiated [5], but in many settings the decision to start therapy is made without any laboratory support, and instead makes use of disease staging criteria [10]. Although early signs of disease can be apparent within a few years of HIV infection [11,12], advanced immune depletion and the need for ART are not always associated with symptoms [13–15].

Whilst a great deal of work has focussed on the optimal state at which to initiate ART [16–19], the way in which this process interacts with other aspects of ART delivery to determine overall efficacy has not received close attention. Understanding the factors that determine the impact of ART programmes will help inform best-practice guidelines and facilitate more accurate projections of future health-care needs. However, making predictions of the effect of ART at the population level is complicated by variability between patients in the rate of disease progression, uncertainty in CD4^+^ cell count measurements, and other stochastic events, such as unrelated causes of mortality and opportunities for diagnosing women during pregnancies. In clinical trials, it would be unethical to compromise patient management and impossible to compare the full range of alternative management strategies. To address these issues, we have developed a mathematical simulation model that follows a theoretical cohort of HIV-infected individuals as the disease progresses, tracking when symptoms occur, how the individual is monitored, the decision to initiate ART, and the effect ART has on survival.

## Methods

### Approach

Our stochastic cohort model represents 1,000 individuals infected with HIV. Each individual is assigned particular characteristics (such as the rapidity of immune suppression and when symptoms occur), and the model calculates when the individual would be expected to die if he or she did not have HIV, had HIV but no treatment, or had HIV and treatment were available. By representing a cohort of individuals with a range of characteristics, we can estimate the mean population level outcomes, such as life-years saved by treatment, and how much variability there is in these outcomes.

The impact of treatment with different strategies for its delivery was analysed by running the cohort model under a variety of assumptions concerning (i) how and when the infection is diagnosed; (ii) how frequently individuals are monitored before they start ART and whether they are lost to follow-up; and (iii) whether CD4^+^ counts are used to help decide when to initiate ART. Since long-term survival on ART was not known, the simulations were repeated making different assumptions about this, based on the short-term observational data that is available [6,7].

Individuals can be diagnosed when they develop symptoms and present at a clinic, or when they are referred from an antenatal clinic (ANC), or when they voluntarily get tested for HIV (voluntary counselling and testing, VCT). For simplicity, we investigated circumstances that range from the worst-case to the achievable best-case scenario. We varied the fraction of women referred from ANC between 10% and 90%, and the fraction of infected individuals that receive VCT whilst they are still healthy between 5% and 70%. Various strategies were designed for monitoring individuals found to be infected but not yet on ART (Table 1). The scheduled intervals between appointment were set at 24, 12, 6 or 3 mo, and could be the same for all patients or vary according to the patient's age and/or degree of immune suppression. We assumed that the fraction of individuals “lost to follow-up” each year at this stage varies between 15% and 0%. Different decision rules for initiating ART were also incorporated in the model (Table 2). With these different rules, the influence of ART being initiated with and without CD4^+^ cell counts, or with different CD4^+^ cell count thresholds, was investigated.

**Table 1:**
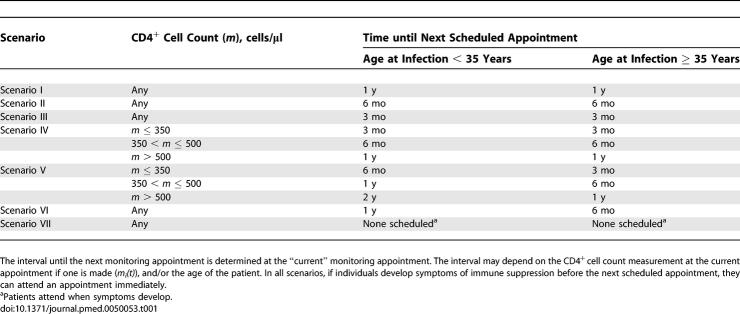
Possible Monitoring Strategies for HIV-Infected Patients prior to Starting ART

**Table 2:**
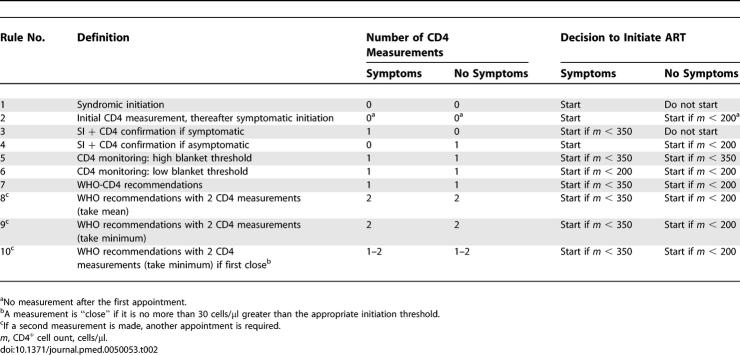
Possible ART Initiation Rules Used in the Model

Our analysis had three parts. First, we compared individual and population outcomes when there is no ART with the situation when ART is available but delivered suboptimally (i.e., few individuals enter care early, monitoring is infrequent, and no CD4 cell counts are taken) and with the situation when some elements of ART delivery are improved. Next, we looked at the impact of these strategies on the health-care system and identified how observed trends in survival or efficiency may be affected. Finally, we compared the different monitoring (Table 1) and ART initiation strategies (Table 2) in order to inform decisions on how ART delivery could make best use of available resources.

### Technical Specification of Model and Data Sources

The mathematical model stochastically simulates disease progression in a cohort of 1,000 HIV-infected adults and tracks the services they receive and key health indicator outcomes. Each individual is realised independently and the properties of the individual and the timing of events are calculated probabilistically based on a series of rules and parametric distributions. Draws from the distributions are simulated by transforming standard uniform deviates from the pseudorandom number generator in MATLAB software (version 7.0.1.24704 (R14) Service Pack 1; Mathworks). The data used to parameterise the disease-progression part of the model were taken from several African studies, and the composition of the model cohort was based on the age and sex distribution of incident HIV infections between 1998 and 2002 in eastern Zimbabwe [20]. Life expectancy in the absence of HIV was calculated using observed mortality rates among uninfected individuals [21].

Each individual was assigned a CD4^+^ cell count after seroconversion and a fixed rate of CD4^+^ cell decline [22], so that in the cohort there was a range of fast and slow progressors. A steady decline in the square-root of CD4^+^ cell count is theoretically [23], clinically [24], and statistically [25–27] justified. In the model, older individuals (> 35 y) progress to immune suppression faster than younger individuals. The CD4^+^ cell count at which an individual develops symptoms sufficiently severe to seek medical attention was drawn stochastically from a distribution based on data from clinic attendees in Côte d'Ivoire [28].

After the CD4^+^ cell count reaches 200/μl, the time until death without treatment was exponentially distributed with a median 11 mo [29]. The median time between HIV infection and death in the model was ∼9.5 y, which is in good agreement with independent observation [30]. The parameters determining the CD4^+^ cell count at which clinical signs of severe immune suppression may be detected by a trained clinician (WHO stage 3 or 4) was based on data from Uganda [13] and Ethiopia [14].

A range of possible points at which an individual could be diagnosed with HIV is represented in the model. Individuals could discover they are infected when presenting at a clinic after developing symptoms. Other individuals could find they are infected when tested at an ANC or attending VCT. Age- and disease state-specific fertility rates [31–33] were used to capture the timing of pregnancies, and two scenarios are defined for the chance that a pregnant women attends an ANC and is referred to the ART programme: low referral rate (10% of pregnant women) and high referral rate (90%). In addition, VCT uptake can be low (5% of individuals receive VCT) or high (70% receive VCT). Alternatively, the individual may die before being diagnosed with HIV.

Once individuals are known to be infected with HIV, they are then managed by the hypothetical “ART programme.” Immediately after diagnosis there was an “appointment” in which the need for ART was assessed. If the individual did not start ART at the first appointment, another was scheduled after a set interval; in the model there were seven possible scenarios, labelled I to VII (Table 1). Some individuals (selected randomly) may drop out at follow-up and not return for further scheduled monitoring appointments. If any patient developed symptoms before the next appointment, they nevertheless attended another appointment immediately. Whether an individual should start ART was determined by the initiation rule. This rule could be based on symptoms and/or CD4^+^ cell count measurements. There were nine possible initiation rules in the model, numbered 1 to 9 (Table 2). Each measurement of the CD4^+^ cell count was assumed to be subject to random error, to reflect short-term physiological variation and technical laboratory factors [34,35].

In the absence of long-term follow-up studies from low-income settings, three sets of assumptions were made about survival on ART termed “best”, “medium,” and “worst”. First-year mortality was parameterised using data from the ART-LINC collaboration of cohort studies in low-income settings [7], with the medium scenario set by the point estimates and the other scenarios set by the bounds of the 95% confidence intervals. The relationship between CD4^+^ cell count, symptoms, and hazard of death after the first year was based on data from high-income settings [6]. In the “best” scenario, the hazard of mortality observed in the first 3 y was assumed to stay constant over time on ART; in the medium scenario it increased gradually; and in the pessimistic scenario the hazard of death increased sharply. The “medium” scenario, which was used in simulations unless otherwise stated, produced 4-y survival rates of ∼75% for those starting with CD4^+^ cell count below 50 cells/μl, and 90% for those starting with CD4^+^ cell count between 200 and 349, which is in good agreement with longer-term analyses of the ART-LINC cohort data [36].

Pregnant women who were in care or attended an ANC during pregnancy were eligible to receive treatment to prevent mother-to-child transmission if they were not already on ART. The model was parameterised to reflect mother-to-child transmission in the context of treatment using a single dose of nevirapine, followed by 7–17 mo of breast-feeding [37,38]. Child deaths were defined as deaths before the 15th birthday, and a child was assumed to be (maternally) orphaned if the mother died whilst they were alive and before the 18th birthday. The number of days that individuals spent with symptoms (“sick days”) was used as a measure of morbidity.

Further details of the model and data sources are given in Text S1.

## Results

### The Impact of Alternative Strategies on Patient Outcomes

The model predicts that in the absence of treatment, infected individuals will each lose, on average, ∼22 y of life (± two standard deviations from mean over 20 stochastic runs: 21.0–23.0 y) and die aged 39 y (38.2–39.8 y) years having experienced severe symptoms for ∼800 d (750–850 d) (Table 3).

**Table 3:**
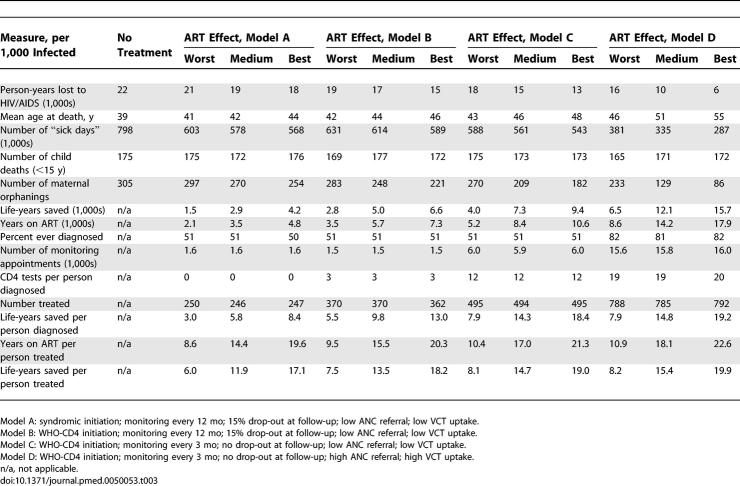
Key Indicator Outcomes for Alternative Initiation Decision Rules and Health-Care System Parameters

**Figure 1:**
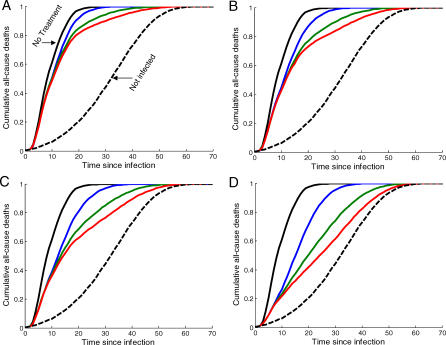
Survival Distribution of an Infected Cohort in Different Models Solid black line, no treatment is available. Where treatment is available, the blue line indicates that ART is available and its assumed effect is worst, the green line indicates a middle effect, and a red line indicates the effect is the best. The survival of an age and gender-matched cohort that is not infected is shown for comparison (dashed black line). The parameterisations of the ART programmes are the same as in Table 3: (A) Syndromic initiation (rule 1), monitored every 12 mo, 15% drop-out, low ANC referral, and low VCT uptake; (B) CD4 initiation (rule 7), monitored every 12 mo, 15% drop-out, low ANC referral, low VCT uptake; (C) CD4 initiation; monitored every 3 mo, no drop-out; low ANC referral, and low VCT uptake; (D) CD4 initiation, monitored every 3 mo, no drop-out, high ANC referral, and high VCT uptake.

If patients are initiated on ART only when they develop symptoms of immune suppression (syndromic initiation), referral of infected women from ANC to the ART programme is low, and uptake of VCT is low, then the predicted impact of ART on these population-level indicators of mortality and morbidity is modest (model A in Table 3). Although the lives of those treated is extended by 6–17 y (depending upon the effect of ART assumed), the average life-years saved among all those infected (treated or not) is equivalent to only 2–4 y per person. The effect of ART is limited by the failure to diagnose many individuals and by starting treatment late when the immune system is already weakened. Cumulative mortality in the first few years of infection is similar to the scenario in which treatment is not available, because most fast progressors die without being treated, and the opportunity to save life-years would be available only for those with advanced disease (Figure 1A).

A CD4^+^ cell count can provide an early warning of immune system depletion before symptoms develop. When a CD4^+^ cell count is used to help decide when to initiate ART in the manner recommended by WHO [5], ∼50% more individuals are started on ART and the extension of life for those treated rises somewhat, to between 8 and 18 y (Figure 1B; model B in Table 3).

Our model shows that the frequency with which patients are monitored also determines the impact of ART (Figure 1C; model C in Table 3). With more frequent monitoring there is a better chance that ART can be started at the right time. In model simulations with the same initiation rule, patients who are monitored every 3 mo instead of every 12 mo (and attend all scheduled appointments) are expected to live approximately 1 y longer on treatment (Table 3), but this outcome would also depend on the route through which individuals entered care (Figure A in Text S1).

The effect of the ART delivery programme is further improved if referral from ANC and uptake of VCT is improved so that more individuals are diagnosed and monitored earlier in the course of infection (Figure 1D; model D in Table 3). The model predicts that with high levels of referral from ANC and VCT uptake, ∼80% of HIV-infected individuals would enter care. In this scenario, the total number of life-years saved is almost double that when referral is low. With earlier diagnosis of infection, there are also fewer sick days because clinical signs can be used to initiate ART promptly.

Increasing opportunities for early diagnosis has two benefits: first, it allows more people to enter care and receive ART before they die; and second, it increases the chance that ART can be initiated at the right time. To isolate the second effect, alternative cohorts are compared in which either all individuals enter care through referral (from ANC or VCT) or individuals can enter care only by presenting at a clinic with symptoms. With referral, the CD4^+^ cell count at which ART is started tends to be higher (mean 33 cells/μl), which leads to more life-years saved by ART (∼0.5 y per person treated) (Figure B in Text S1). Referral of women from ANC is especially productive because they are typically young and disease-free (Figure C[a] in Text S1).

**Figure 2:**
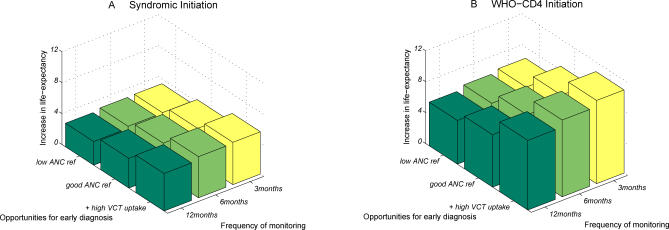
Improvements in Life Expectancy at Infection Due to the Availability of ART In (A) only symptoms are used to initiate ART (rule 1); in (B) one CD4^+^ cell count measurement is used in the way WHO recommend (rule 7). In both panels, 5% yearly drop-out rate is assumed.

Altogether, these three factors—the timing with which ART is initiated, the opportunities for early diagnosis of infection, and the frequency with which patients in care are monitored (without drop-out)—combine to determine the improvement in life expectancy at infection due to the availability of ART (Figure 2). Upgrading from syndromic initiation with low ANC referral rates, low VCT uptake, scheduled monitoring every 12 mo, and 15% of patients dropping out each year, allow ∼60% more individuals to enter care, almost twice as many entering care to be started on ART; furthermore, individuals on ART have longer survival (up from 6 y to 15 y) (Figure D in Text S1). This improvement leads to three times as many HIV-infected individuals starting treatment. In total, this could mean that life expectancy at infection by could be increased from 10 y with no ART (worst-case), to 12–14 y with suboptimal delivery of ART (medium), up to 17–27 y with this “best-case” ART delivery scenario (ranges due to different survival assumptions). The impact of intermediate scenarios for VCT uptake and ANC referral rates were also investigated (Table A in Text S1).

### Impact on the Health-Care System

When more individuals are diagnosed and diagnosed at an earlier stage, the case load for the ART programme is higher (Table 3). The average difference between the worst- and best-case scenarios described above is 14 more appointments per person infected, 19 CD4^+^ cell counts taken per person diagnosed, and 10 y more on ART, per person infected (Table 3). It is therefore essential to implement strategies that maximise patient outcomes whilst minimising costs (years of therapy and time of health-care workers). Recommendations for how this can be done are made in the following section.

However, the model does identify complicating factors in measuring the performance of programmes that should be considered. First, the apparent effectiveness of ART at the population level may not improve when more individuals are diagnosed earlier through VCT (Figure C[b] in Text S1). Those progressing to AIDS fastest, who would otherwise die outside of the ART programme, will start ART when already immune suppressed and die within the programme, bringing down measures such as average survival time on ART. Second, the number of life-years saved per year on ART may decrease if infections are diagnosed earlier, because some patients would start ART too soon (due to random error in CD4^+^ cell measurement or early symptoms), but would otherwise have survived for some years more without treatment. Other patients would enter care who are at an early stage of infection or are “slow progressors,” and these individuals would be monitored unnecessarily for many years.

### Implications for Resource-Poor Settings

**Figure 3:**
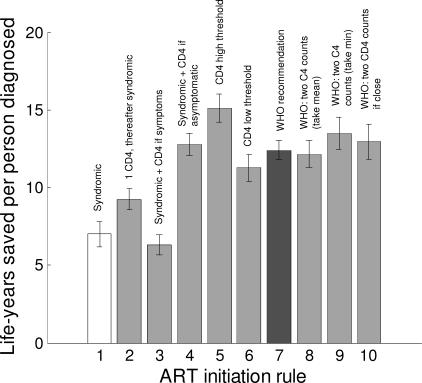
Comparison of Possible Initiation Rules in Years Saved per Person Diagnosed Appointments are scheduled for every 6 mo. Error bars show ± 2 standard deviations from 20 stochastic runs. 5% yearly drop-out rate is assumed. Details of rules are listed in Table 2.

Alternative strategies for ART initiation and patient management (Tables 1 and 2) were investigated. In these simulations we assumed a 5% yearly drop-out rate during follow-up, but the qualitative relationship between the strategies investigated was not influenced by this assumption. In terms of life-years saved per person diagnosed, there is a clear advantage in using CD4^+^ cell counts to check that asymptomatic individuals are not severely immune suppressed (Figure 3: rules 1–3 versus 4–10). Using only one CD4 measurement (taken at the first monitoring appointment) enables substantially more life-years to be saved than does syndromic management alone (rule 2 versus rule 1). The alternative strategies of testing everyone (rule 7), and testing only those without symptoms (rule 4) are similarly effective, although the latter requires ∼10% fewer CD4 measurements (Table B in Text S1). If treatment is initiated at higher CD4^+^ cell levels than WHO recommends (rule 5), more life-years are saved but this timing requires disproportionately more years on ART (years on ART per life-year saved: 1.20 versus 1.17), because many individuals would survive for years after reaching this threshold without ART. The opposite is true if ART is used more selectively by initiating at lower CD4 levels (rule 6); here, fewer life-years are saved but many fewer years are spent on ART (years on ART per life-year saved, 1.11 y).

When all strategies are compared, those that use more CD4 measurements tend to save more life-years overall and more life-years per year on ART. The advantage of CD4^+^ cell counts is greater when the association between CD4 level and symptoms is weak (Figure E in Text S1). However, the model suggests that CD4^+^ cell counts remains advantageous under a variety of scenarios for this relationship (Figure E in Text S1).

The physiological variability of CD4^+^ cell counts means that taking two measurements instead of one could lead to better clinical decision-making for individuals. However, in the model, basing clinical decisions on the mean of two measurements does not lead to substantial improvements, because it is assumed that measurement errors are equally likely to lead to earlier initiation as to later initiation (Figure 3: rule 8). Basing the decision on the minimum of two measurements, in contrast, does increase the chance of ART initiation, and more life-years are saved without a substantial increase in years spent on ART (Figure 3: rule 9). If all patients have two CD4^+^ cell measurements, twice as many monitoring appointments and CD4^+^ cell counts are required, but if a second CD4^+^ cell count is taken only when the first is just above the initiation threshold (within 30 cells/μl), then fewer tests are required per life-years saved (Figure 3: rule 10; Figure F in Text S1).

With more frequent monitoring, the chosen ART initiation strategy can be implemented more accurately and more life-years can be saved (Figure G[a] in Text S1). For the same reason, drop-out from follow-up appointments (patient not attending the next scheduled appointment) can result in substantial reductions in the impact of ART programmes (Figure G[b] in Text S1). Scheduling appointments is essential because relying on individuals to attend only when they experience symptoms defeats the advantages of early diagnosis. Infrequent monitoring and/or high drop-out rates are particularly damaging to programmes using CD4^+^ cell counts because these factors increase the chance that patients are not monitored as they cross the threshold for ART initiation (Figure G in Text S1).

An efficient allocation of appointments is to schedule patients with high CD4^+^ cell counts to be monitored less frequently than those with low CD4^+^ cell counts (scenario IV) (Figure H in Text S1). The model predicts that in this system, there would be 50% more appointments than if everyone were monitored every 12 mo, but this increase would lead to 2.1 more life-years saved per person diagnosed (Figure H in Text S1). The number of appointments is reduced further without a substantial reduction in the life-years saved if the scheduling system also takes account of age (scenario V), where young people are monitored less frequently than older (35+ y) people.

## Discussion

There is great potential for ART to reduce premature deaths due to AIDS in resource-poor settings, but inadequate monitoring of HIV-infected individuals not on treatment could prevent this potential from being fully realised. Our modelling shows that using CD4^+^ cell counts to determine when to initiate ART could greatly increase the number of life-years saved, because it enables individuals to receive ART when the effect of therapy is greatest, before the immune system is severely weakened [6,7]. New CD4^+^ cell counting technology is more affordable and better suited to conditions with limited health-care infrastructure [39–41], and in the “3 by 5” program (the campaign to get 3 million on therapy by 2005) most of the resource-limited focus countries used CD4^+^ cell counts to help judge when ART should be initiated [42].

However, since individuals tend to present at clinics with advanced disease [28], the ART programme must be competent at finding individuals at earlier stages. Regularly monitoring patients can further improve their prognosis because it increases the chance that ART can be started at the right time. Although HIV testing services are being rapidly scaled-up, currently only 8%–25% of those infected have discovered that are living with HIV [43]. However, the movement towards provider-initiated testing [44] is expected to lead to great increases in diagnosis of HIV infections. In Botswana, where a similar policy was implemented in 2004, almost half of a general population sample reported having had an HIV test [45]. Referring women who have tested positive at ANCs is expected to be especially productive, because they are likely to be young and at an early stage of disease.

The CD4^+^ cell count is a more sensitive indication of need for ART than the presence of symptoms, so CD4^+^ cell count-based initiation is expected to enable more life-years to be saved and more life-years saved per year of therapy. The model shows that even making just one CD4 measurement at the first appointment, to catch those in need at ART when they first enter care, is likely to improve the impact of the programme substantially. Testing at every appointment, and basing the clinical decision on two counts for borderline cases, maximise the usefulness of the available CD4^+^ cell count information. Routinely starting patients with higher CD4^+^ cell counts than the WHO recommends would save more life-years overall, but fewer life-years per years on therapy. Avoiding CD4^+^ cell counts for those who have already developed symptoms could reduce the number of counts required without sacrificing impact, because few symptomatic individuals will have a CD4^+^ cell count higher than 350 cells/μl.

The efficiency of appointment scheduling can be improved by prioritising the more immune suppressed (CD4^+^ cell count ≤ 350) and older (35+ y) patients, since they probably need ART sooner. Relying on individuals to return to the clinic when they develop symptoms would reduce the number of appointments required, but is not an effective way to manage patients; symptoms unreliably predict the need for ART.

This model analysis shows that increases in rates of patient referral, earlier and more frequent monitoring of HIV-infected patients, and better rules for initiating ART could lead to increases in the number of appointments with ART providers and the amount of ART required. By evaluating the cost of providing these services, a cost-effectiveness study could determine, for a specific location, which of these changes would lead to the most efficient allocation of resources [46]. Without considering the costs involved, it is not possible to make specific recommendations about the optimal method for managing patients.

The substantial differences in expected ART outcomes for different programmes and modes of patient management should lead, over time, to large differences in the number in need of therapy. Projections of ART requirements will therefore need to examine how ART is initiated and how patterns of diagnosis and referral could change. Current estimates that are based on calculating the fraction of infected individuals in the last few years before death do not take the variation in strategies or their evolution over time into consideration [38,47,48].

The model is limited by a lack of data on the relationship between the WHO staging criteria and CD4^+^ cell count, which underlies the quantitative estimates of the benefit of different types of initiation. Although the average CD4^+^ cell count among patients with certain conditions has been reported in several studies [11,12,49], it is not possible to know which of these patients a clinician would determine to be in need of ART. The data we have used to parameterise the model suggest that many individuals with low CD4^+^ cell count would not be categorized as appropriate for ART under WHO guidelines [13,14]. However, our analysis indicates that monitoring CD4^+^ cell counts remains generally advisable under a wide range of scenarios for this relationship, including the development of symptoms before severe immune suppression in the majority of people (Figure E in Text S1).

In the absence of more detailed data, the model does not differentiate between WHO stage 3 and 4 disease and cannot replicate the clinical judgement that should be used to determine how soon patients with stage 3 symptoms should be started on ART [5]. Instead it identifies individuals who develop “severe symptoms of immune suppression” that are analogous to WHO stage 3, and we explore the effects of using different CD4^+^ cell count thresholds for starting ART. Under the WHO recommendations, patients with stage 4 symptoms should be started on ART regardless of their CD4 level, but in the model they are started only if their CD4^+^ cell count falls below the threshold being used for starting any symptomatic patient. However, patients with stage 4 disease seldom have higher CD4^+^ cell counts than this, so the model will only slightly underestimate the impact of following the WHO guidelines.

A public-health approach to delivering ART has to consider how to initiate ART and organise the delivery programme to maximise the benefit to the population overall. According to this model, diagnosing infections earlier (through referrals from ANC or VCT), regularly monitoring patients, and using CD4^+^ cell counts to initiate ART will save more life-years. Unless this care is available to patients at all stages of HIV infection, the long-awaited chance to substantially reduce AIDS mortality with ART could fall far short of its full potential.

## Supporting Information

Text S1 Extended Methods Section and Further Results (274 KB PDF)

## Abbreviations

ANC: antenatal clinic
ART: antiretroviral treatment
VCT: voluntary counselling and testing
