# Supplementary material for: The Impact of Monitoring HIV Patients Prior to Treatment in Resource-Poor Settings: Insights from Mathematical Modelling
Source: PLoS Med. 2008 Mar 11;5(3):e53. doi: 10.1371/journal.pmed.0050053 (PMC2265759; doi:10.1371/journal.pmed.0050053)
Supplement: Supplementary file 1 [file pmed.0050053.sd001.pdf]

The impact of monitoring HIV-infected patients prior to treatment in Resource-Poor Settings: Insights from Mathematical Modelling

Supplementary Material

1. Extended Methods Section (Figures 1-5 & Tables 1-3)

2. Further Results: Eight figures and two tables (Figures A-H & Tables A-B)

1. Extended Methods Section

The model stochastically simulates the progression to AIDS and death in a cohort of HIV-infected individuals and tracks the services they receive and selected health indicator outcomes. Each individual is realised independently and the properties of the individual and the timing of events are calculated probabilistically based on a series of rules and parametric distributions. The data used to parameterise the disease-progression part of the model are taken from several African studies, and it was decided that the characteristics of the cohort would reflect the current epidemiological conditions in eastern Zimbabwe.

Characteristics of the cohort

The composition of the cohort is based on the gender and age distribution of newly infected individuals in the Manicaland cohort study, situated in the eastern highlands of Zimbabwe, between 1998 and 2002[1] (Table 1). To capture a suitable range of stochastic effects, the number of individuals in the cohort for most simulations is 1000; the effects described in this paper and not sensitive to the size of the cohort. Mortality from non-HIV/AIDS related causes among children is assumed to be: 53 per 1000 live births for infants (0-1years), and a total of 90 per 1000 live births die before their fifth birthday (deaths distributed exponentially with parameters 0.054 for 0-1 year-olds, and 0.010 for 1-4 year-olds)[2]. The chance of dying between ages 5 and 99 is Weibull distributed such that median life-expectancy in the absence of HIV/AIDS is 48.8 years for men and 52.5 years for women, as observed in the

Manicaland cohort for uninfected individuals[3] (shape and scale parameters for males: 7 and 57.5; for females 7 and 61.8).

Age group	Males	Females
**15-19**	0.048	0.111
**20-24**	0.095	0.146
**25-29**	0.100	0.148
**30-34**	0.089	0.032
**35-39**	0.084	0.026
**40-44**	0.025	0.016
**45-49**	0.033	0.013
**50-54**	0.034	0.000

Table 1 Distribution of incident infections with respect to age and gender, based on incident infection in Manicaland 1998-2002[1].

Clinical progression

For the ith individual, the CD4 count ($CD4_i(t)$) declines over time since infection (t) according to the function:

$$\begin{aligned} &\sqrt{CD4_i(t)} = a_i - b_i t \\ &a_i \sim N(\mu_a, \sigma_a) \\ &b_i \sim N(\mu_b^0, \sigma_b) \quad \}\text{if infected when age} < 35 \text{ years} \\ &b_i \sim N(\mu_b^1, \sigma_b) \quad \}\text{if infected when age} \geq 35 \text{ years} \end{aligned} \quad \ldots(1)$$

The unit of $CD4_i(t)$ is number of cells per microlitre of peripheral blood $(\mu l)^{-1}$. A steady decline in square-root CD4 is theoretically[4] and clinically[5] justified and uses parameters that are directly comparable to those in evaluated in several statistical analyses[6,7,8]. Although this is not likely to be a good description of CD4 decline immediately after infection, it should be sufficient for our analyses, which concern using CD4 count information once the infection is established.

The values of $\mu_a = 25.91, \sigma_a = 0.61$ and $\mu_b^0 = 1.32$ are taken from a study of populations in Western and West-Central Africa[9]. The value $\sigma_b = 1$ matches a Bayesian shrinkage estimate[7] of the variance in the rate of decline of CD4 counts. This means that in the model 68% of individuals younger than 35 years are assigned rates of CD4 decline within one standard deviation of the mean (0.3 to 2.3) and of the rest, half are assigned a rate of decline less than 0.3 (slow-progressors) and half are

assigned a rate of decline greater than 2.3 (fast-progressors) (Figure 1(a)). The value of $\mu_b^1 = 2.0$ is chosen to reproduce shorter survival times for older people[10,11].

Each measurement of CD4 count is modelled as an observation of $CD4_i(t)$ plus random error (with standard deviation σ_m). The error derives partly from technical factors[12], but mostly from short-timescale physiological fluctuations in the true CD4 count[13,14,15]. In laboratory studies where counts have been repeated on the same blood sample and on blood samples taken within the same few weeks, the combined variation is estimated to have a standard deviation of approximately 50. In the model, it is assumed that this variation is distributed uniformly, with mean zero. The value upon which the clinical decision is taken ($m_i(t)$) can be a function of several $(1,2,..j,..,n)$ measurements that are taken on separate occasions. Unless otherwise stated, in all analyses $n=1$, but when initiation rules 8 or 9 are used (Table 2 in main text) $n=2$ and f is the mean or the minimum.

$$\begin{aligned} &\varepsilon_j \sim U(-\sqrt{3}\sigma_m, +\sqrt{3}\sigma_m) \\ &m_i(t) = CD4_i(t) + f(\varepsilon_1, \varepsilon_2, ...\varepsilon_n) \end{aligned} \qquad \text{...(2)}$$

Figure 1(b) show successive CD4 measurements for five randomly-chosen individuals.

(**a**)

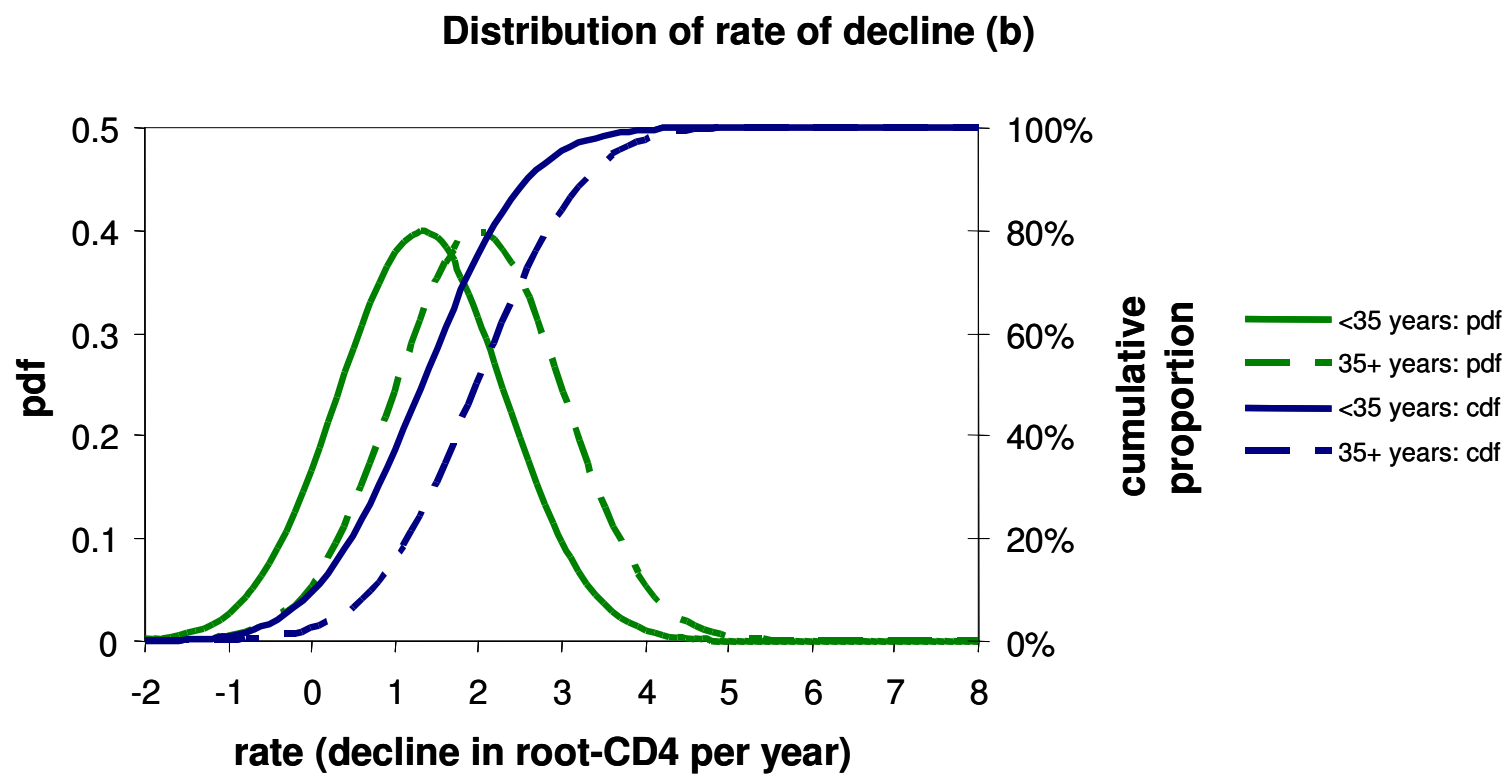

(**b**)

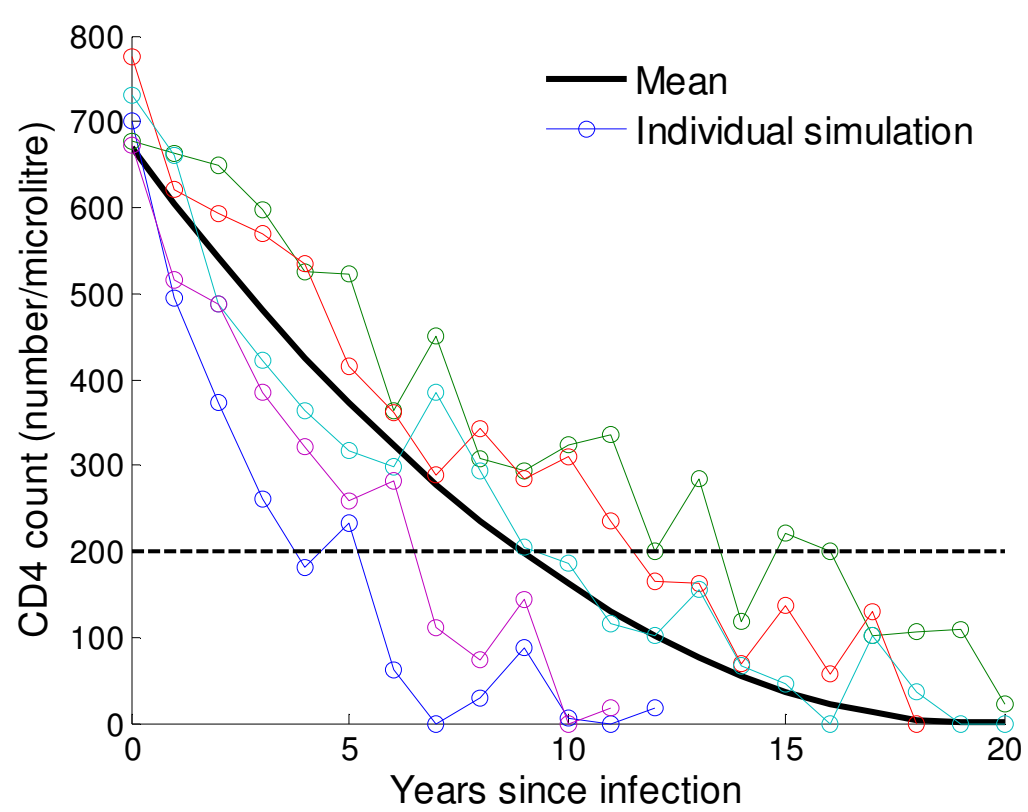

Figure 1 (a) The distribution of rate of decline of CD4 count (square-root transform of value in number per microlitre) for those infected when younger than 35 years (solid lines) and when 35 years or older (dashed lines). Green line (left axis) give probability density function (pdf) and blue line (right axis) give cumulative distribution. (**b**) Change in CD4 count over time since infection. Thick line gives mean trend and other lines show the progression of five randomly-chosen individuals observed once a year.

The relationship between clinical signs (WHO stage III or IV) and immune-suppression is not well characterised. To incorporate this uncertainty in the model, three alternative scenarios were defined (Figure 2). Scenario I (which is used, unless specified otherwise) is based on observations from Uganda[16] and Ethiopia[17] (Double Weibull distribution with first shape and scale parameters 2.2 and 80.0 and second shape and scale parameters 12.4 and 399.3, respectively, weighting to first 0.53). In scenario II 90% of individuals develop symptoms before their CD4 count drops below $200(\mu l)^{-1}$ (Weibull distribution shape parameter 3, scale parameter 355), and in scenario III 15% of individuals develop symptoms before their CD4 count drops below $200(\mu l)^{-1}$ (Weibull distribution shape parameter 1.5, scale parameter 130).

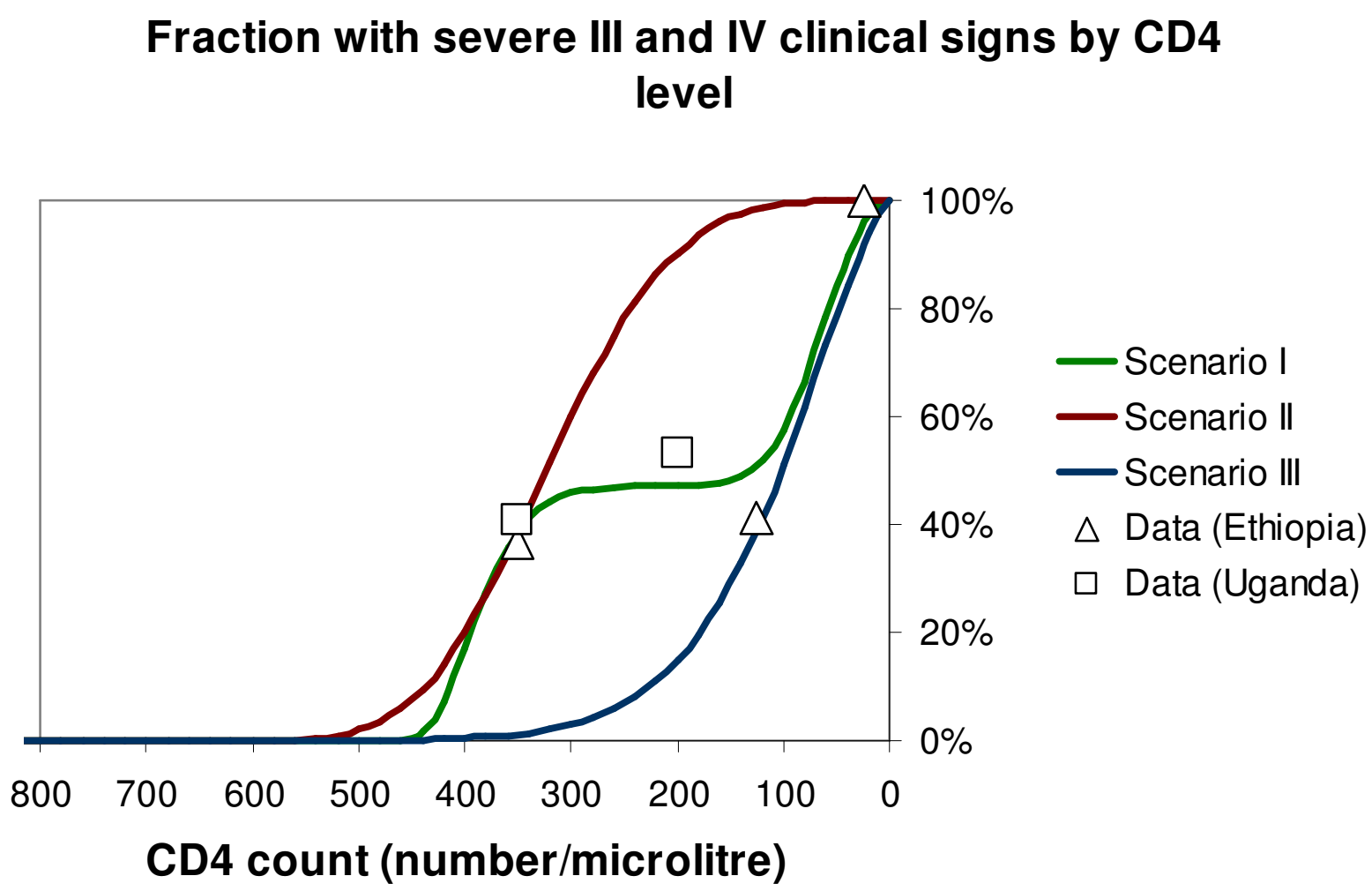

Figure 2 Scenario I (green line) and scenario II (red line) for CD4 level when symptoms of severe symptoms of immune-suppression (WHO stage III and IV) develop. Squares show data points from a study in Uganda[16] and triangles show data points from a study in Ethiopia[17].

Survival after CD4 count falls below 200 $(\mu l)^{-1}$ is assumed to be exponentially distributed with a median survival of 11 months, in accordance with a review of studies from resource-poor settings[18].

Survival from infection to death for young individuals has been estimated to be 10.9 years in Western populations[10] and 9.8 years in Uganda[11], although some studies have indicated lower survival rates in other African populations (reviewed by Jaffar *et al.*[19]). Despite being based on independent data sources, the model is in good agreement, generating a median survival of ~9.5years for individuals infected when younger than 35 years (Figure 3). The shape of the survival distribution for those surviving ten years or more after infection has not been directly observed. In order that the model survival curve approximates a Weibull distribution, the number of individuals with very long survival times is limited by accelerating the rate of progression if they have not developed AIDS after ten years. This is done by replacing the rate of progression (b_i) with a new stochastically sampled value from the same probability distribution, provided it is at least as great as the initial value. In the model the median survival time for individuals infected when they are 35 years or older is ~7 years.

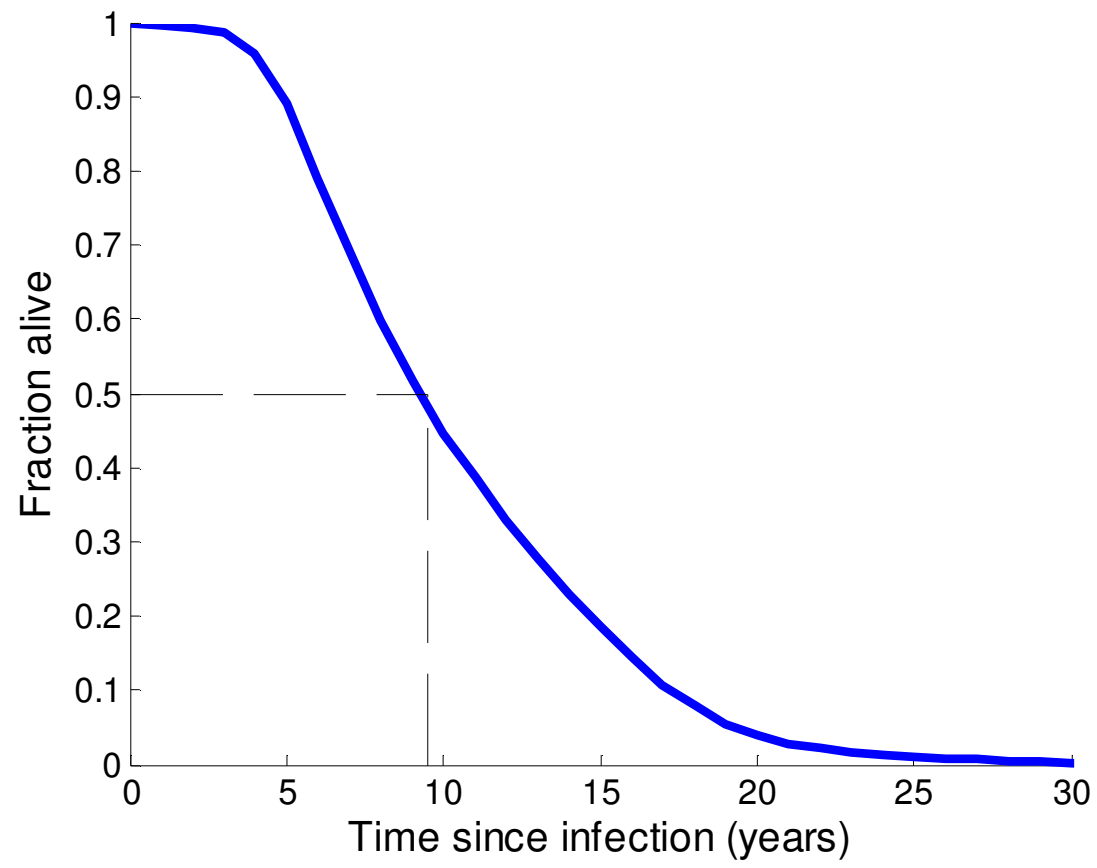

Figure 3 Modelled survival distribution for individuals infected with less than 35 years old, in the absence of treatment (median 9.5 years).

Diagnosis of infection

Individuals may be diagnosed with HIV in one of three ways, whichever comes first. Once the infection is diagnosed, clinical monitoring of the individual can begin (see below). If none of these events happen, the individual is not diagnosed and therefore does not enter care and cannot start treatment.

1. Individual falls pregnant and attends ante-natal clinic (ANC).

   Recent estimates of fertility rates in Zimbabwe[20,21] and measurements of sub-fertility over the course of infection[22] are used to capture patterns of ANC attendance (Table 2). For each pregnancy, the probability that the women will attend an ante-natal clinic, have her infection diagnosed and be referred to the anti-retroviral treatment (ART) programme can be 10% ("poor referral" scenario; estimated to currently be the case in Zimbabwe) or 90% ("good referral" scenario). It is assumed that attendance at ANC is exactly six months before delivery.

2. Individuals go for voluntary counselling and testing (VCT).

   It is assumed that the probability that an individual goes for VCT is constant over time. "Low VCT uptake" means that 5% of individuals (randomly selected) go for VCT and "High VCT uptake" means that 70% of individuals go for VCT. Individuals go for VCT at some time (uniformly distributed) before they would otherwise present at a clinic (see below). It is assumed that individuals that do not go for VCT are either geographically isolated from

services or unwilling to attend for other reasons. High VCT uptake could be achieved following scale-up of services{Population Services International, 2006 #261}, including home-based testing[24], or if policies are changed to allow routine 'opt-out' testing at all medical facilities[25,26].

3. Individual develops symptoms and goes to a clinic

The CD4 level at which an individual develops symptoms sufficiently severe to seek medical attention is determined by when the depletion of the immune-system reaches a predetermined threshold. This threshold varies between individuals and is drawn stochastically from a distribution based on the CD4 levels of those attending a clinic in Cote d'Ivoire[27]: Weibull distribution, shape parameters 0.8, scale parameter 194.1 (Figure 4). Two alternative scenarios are also included, whereby individuals present at the clinic earlier (e.g. in response to minor symptoms[28,29]: Weibull distribution, shape parameters 2.2, scale parameter 400.0) or later (Weibull distribution, shape parameters 0.9, scale parameter 80.0).

Age group	Fertility rate in general population†* (1999 DHS survey[20])	HIV Prevalence (2002)[30]	Average sub-fertility[21]	Fertility of uninfected women†!!	Fertility for infected women, by years before AIDS[22]§			
					4+ (RR=0.79)	2-3 (RR=0.45)	0-1 (RR=0.27)	AIDS (RR=0.19)
**15-19**	112	0.15	0.84	115	91	52	31	22
**20-24**	199	0.25	0.85	207	163	93	56	39
**25-29**	180	0.36	0.78	195	154	88	53	37
**30-34**	135	0.33	0.65	153	121	69	41	29
**35-39**	108	0.31	0.63	122	96	55	33	23
**40-44**	46	0.21	0.65	50	39	22	13	9
**45-49**	15	*0.15‡*	0.65	16	13	7	4	3

Table 2 Assumed fertility of HIV-infected women in the model cohort. †Fertility rate expressed as number of live births per 1000 women per year. *These rates are based on all women in Zimbabwe, some of whom will be infected. ‡ Data not available for this age-group: value interpolated. !!Fertility of uninfected women calculated assuming that observed fertility in DHS survey is composed of infected and uninfected women in proportion to HIV-prevalence at nationally representative antenatal clinics in 2002, and that sub-fertility among all infected-women is as measured in rural areas between 1995 and 2000. §Fertility has been shown to decrease with progressing infection[22]. RR is the observed ratio of fertility of infected women relative to uninfected women in Uganda, and this was used to estimate fertility of infected women in Zimbabwe for the model. In the model, AIDS is defined as when CD4 count falls below 200. It is assumed that women receiving treatment have the same fertility as women with AIDS.

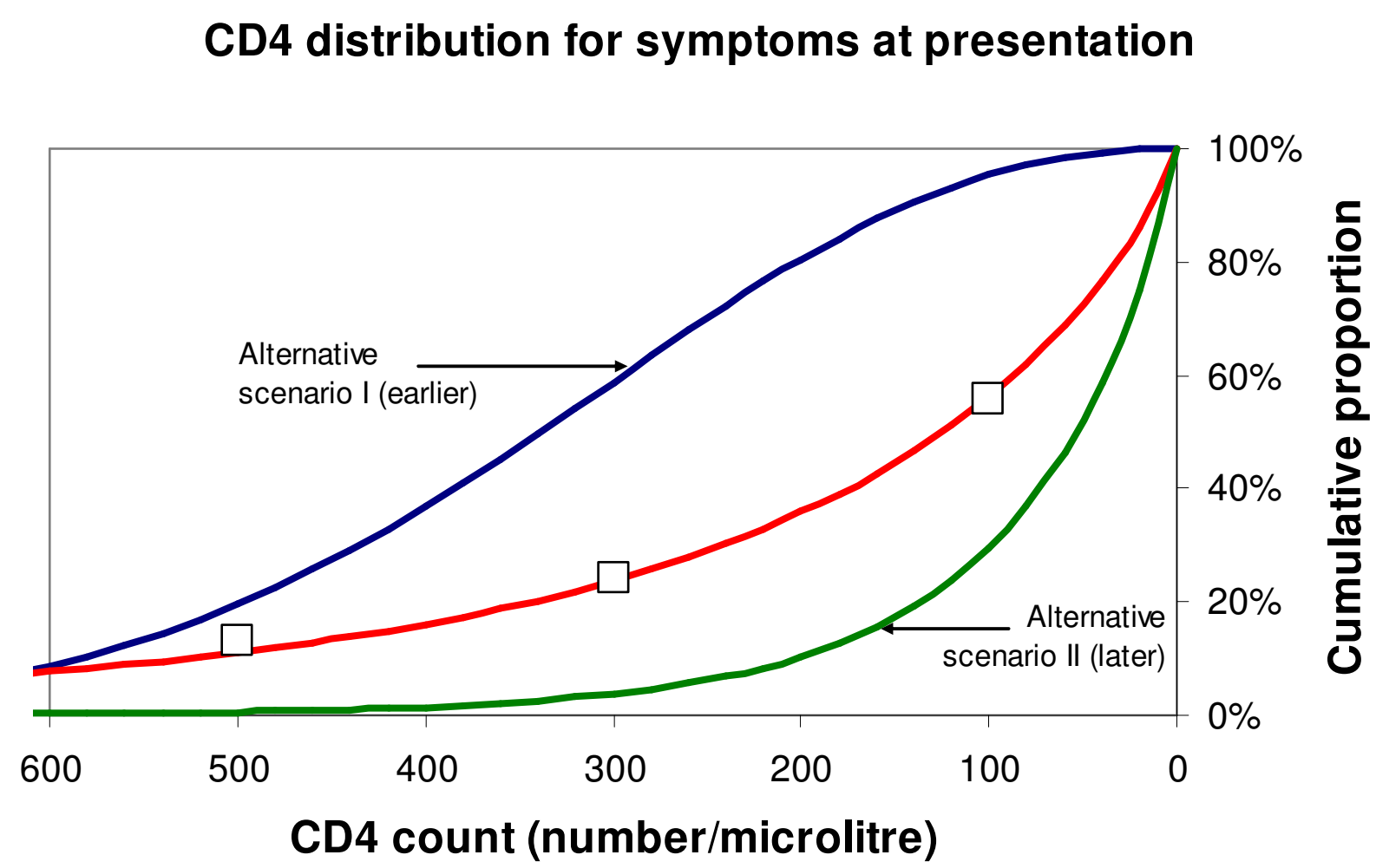

Figure 4 The CD4 level at which individuals will present at a clinic if they have not already been diagnosed. The red line is the default scenario and is based on CD4 counts among those presenting at a clinic in Cote d'Ivoire[27] (white squares). The blue and the green lines are alternative scenarios, whereby individuals present at a clinic earlier or later, respectively.

Clinical Observation

Once the HIV infection is diagnosed, individuals can visit the ART provider (usually a doctor or nurse at a local or district-level clinic) so that their need for ART can be assessed. It is assumed that when an individual is first diagnosed (whether at ANC, VCT or when they develop symptoms sufficiently severe to seek care) they immediately visit the ART provider. At each visit to the ART provider, the date of the next appointment is arranged. The time to the next appointment can be determined by the CD4 count and/or age of the individual; seven different scenarios are used in the model (Table 1 in the main text). Individuals may be lost-to-follow-up (e.g. due to out-migration, incapacity, forgetting etc.) and will not attend the next (or any subsequent) appointment. The assumed rate of loss is constant over time, so that for a set rate (e.g. 0.20), ~18% are lost in the first year, and of those that remain, 15% are lost the next year, then 12% in the year after, and so on. For most simulations, this rate is set to zero.

Decision to Start ART

At each visit to the ART provider a decision is taken about whether the individual will start ART. The algorithm is to start ART if certain criteria regarding clinical symptoms and CD4 measurements are met. A range of algorithms are used in the model (Table 2 in the main text). The decision to start ART can be based entirely on whether or not

the patient has severe symptoms ("syndromic initiation": code 1). The recommendations of the WHO[31] are to start ART if the CD4 count is below $200(\mu l)^{-1}$, or below $350(\mu l)^{-1}$ if the patient already has severe symptoms (code 7). The use of CD4 counts can be conditional on whether the individual has symptoms (codes 2, 3 and 4) and different thresholds for initiation can be used (codes 4 and 5). If an additional CD4 count is taken (codes 8 and 9), then another appointment is scheduled for within a few weeks, and the decision to start ART is taken at the second appointment.

The effect of ART

When starting ART, an individual is assigned a duration of time until they die as a result of treatment failure and/or AIDS (in the absence of other competing causes of mortality not related to HIV/AIDS). A meta-analysis of cohort studies from high-income settings (ART-Cohort Collaboration: http://www.art-cohort-collaboration.org/) has quantified three-year survival for individuals starting therapy, stratified by baseline CD4 and the presence of WHO stage III/IV symptoms[32]. The ART-LINC collaboration of cohorts also finds evidence that the risk of death is higher in low-income settings in the first few months of ART, but similar to the rate in high-income countries later[33]. In the absence of a systematic aggregation of survival rates over a longer period from African cohorts, three survival scenarios were defined ("best", "medium" and "worst"). These scenarios are parameterised in the following way:

i. First-year survival is equal to aggregated estimates from low-income settings[33]. The "medium" scenario uses the point estimate and the "best" and "worst" scenarios use the limits of the 95% confidence intervals.
ii. The relationship between CD4 count, symptoms and hazard of death after the first year is based on data from high-income countries[32]. In the "best" scenario, the hazard of mortality observed in the first three years of therapy is assumed to stay constant; in the default scenario it increases gradually; and in the pessimistic scenario the hazard of death increases sharply with time on ART.

Survival time is Weibull distributed for all scenarios (Figure 5). The parameters for the survival time scenarios are given in Table 3.

The "medium" scenario, which is used in simulations unless otherwise stated, produces four-year survival rates of ~75% for those starting with CD4 count below 50 and 90% for those starting with CD4 count between 200 and 349, which is in good agreement with longer-term analyses of the ART-LINC cohort data, which have not yet been published[34].

WHO stage III/IV & high viral load at baseline	CD4 count at baseline*	"Best" scenario			"Medium" Scenario**			"Worst" Scenario		
		Shape	Scale	First-year failure	Shape	Scale	First-year failure	Shape	Scale	First-year failure
Yes	0-50	1.0	34.2	8.7%	1.6	13.7	10.9%	2.5	7.9	12.1%
	50-99	1.0	43.7	5.3%	1.6	16.0	6.7%	2.5	8.8	7.4%
	100-199	1.0	47.8	3.6%	1.6	16.9	4.6%	2.5	9.1	5.1%
	200-349	1.0	79.7	1.4%	1.6	23.3	1.7%	2.5	11.1	1.9%
	350+	1.0	141.0	1.4%	1.6	33.3	1.7%	2.5	14.0	1.9%
No	0-50	1.0	85.86	8.7%	1.6	24.4	10.9%	2.5	11.5	12.1%
	50-99	1.0	109.70	5.3%	1.6	28.4	6.7%	2.5	12.7	7.4%
	100-199	1.0	119.92	3.6%	1.6	30.1	4.6%	2.5	13.1	5.1%
	200-349	1.0	199.97	1.4%	1.6	41.4	1.7%	2.5	16.1	1.9%
	350+	1.0	353.84	1.4%	1.6	59.1	1.7%	2.5	20.2	1.9%

Table 3 Parameters for Weibull distribution of survival time from start of ART to death. These rates do not include mortality from causes unrelated to HIV/AIDS, which is included in the model as an independent competing hazard. *Number per microlitre of peripheral blood. **Medium scenario is used unless otherwise stated.

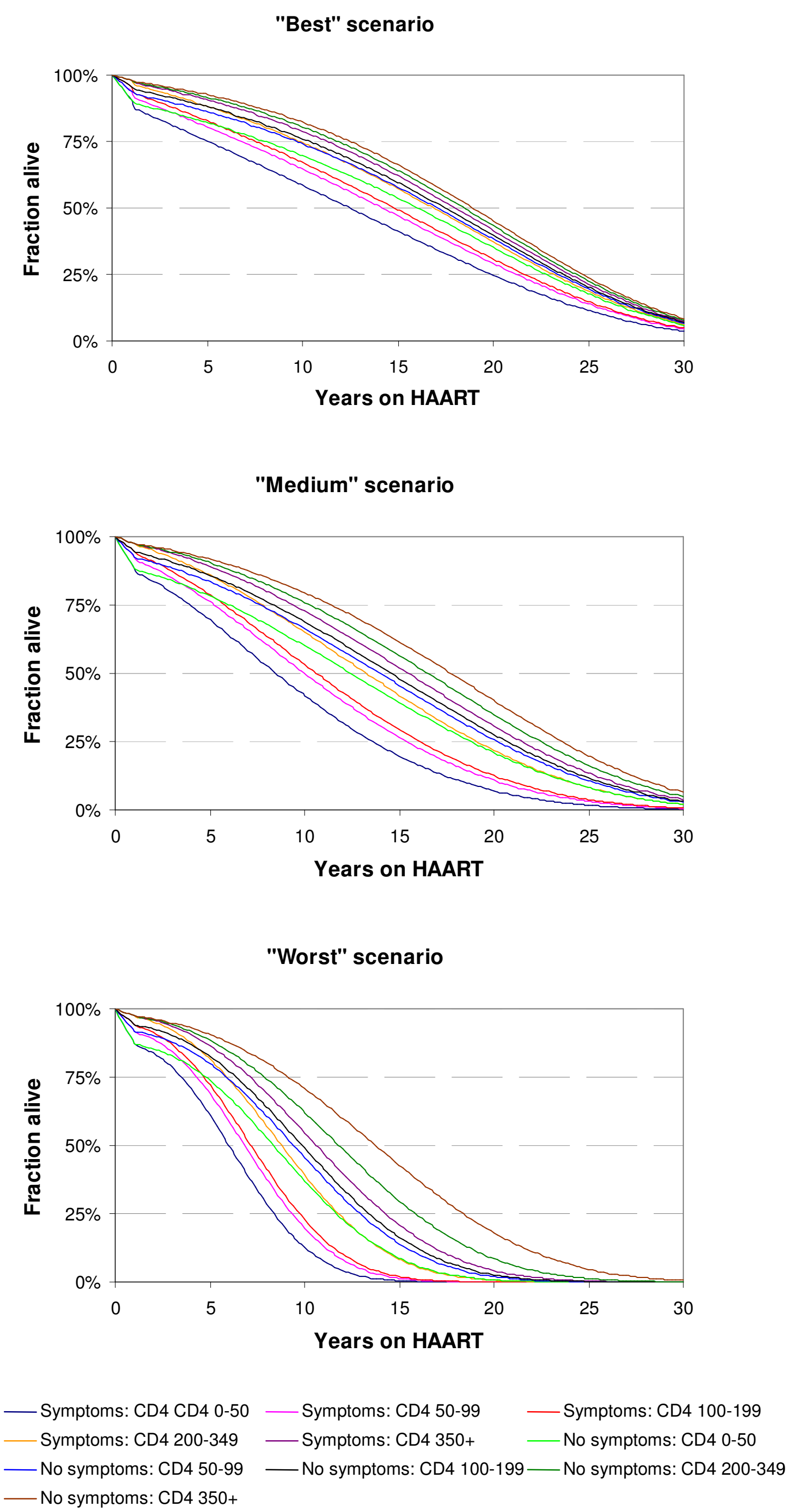

Figure 5 Survival from start of ART with “best”, “medium” and “worst” assumptions, by presence or absence of symptoms and CD4 count at start of ART. Background survival is included for a male starting ART aged 35 years.

Pregnancy and mother-to-child transmission

Pregnant women that have had their infection diagnosed and have not been lost to follow-up are eligible to receive treatment to prevent mother-to-child transmission (PMTCT) if they are not already on ART. In Zimbabwe, it is estimated[2] that the probability that a women is able to receive treatment to prevent mother-to-child transmission is 30%. The probability that a woman accepts to receive therapy under these circumstances is estimated to be 50%.

In the model, the probability that a baby is infected by its HIV-positive mother is 24% if she has received PMTCT, and 32% if she has not. This approximates the case for 7-17 months of breastfeeding and treatment being a single dose of Nevirapine[35,36]. The probability that a baby is infected from its mother who is permanently on ART is 4%[36].

Following the recommendations of UNAIDS[36,37], in the model the additional mortality hazard for an infected baby in the absence of treatment is taken to be distributed as a double-Weibull (first shape and scale parameters 0.97 and 1.52, second shape and scale parameters 5.39 and 10, respectively, weighting to first 0.65). This distribution has two components; the first represents the fast progressors, who are infected in-utero and intrapartum; the second represents the slow progressors, who are infected during breast feeding.

A "child death" is counted if they die before their 15th birthday. A child is assumed to be (maternally) orphaned if their mother dies whilst they are alive and before their eighteenth birthday.

Distribution Notation

If U_i are uniform random deviates between 0 and 1, then:

- Q_i are uniformly distributed between a and b, where:

$$Q_i = a + (b-a)U_i \qquad \ldots(3)$$

- E_i are exponentially distributed with mean μ, where:

$$E_i = \frac{\ln(U_i)}{-\mu} \qquad \ldots(4)$$

- W_i are Weibull distributed with shape parameter k and scale parameter λ, where:

$$W_i = \lambda\left(-\ln(U_i)\right)^{1/k} \qquad \ldots(5)$$

- D_i are distributed as a double Weibull with first shape and scale parameters k_1 and λ_1, second shape and scale parameters, k_2 and λ_2, and weighting w, where:

$$D_i = \lambda_1 \left(-\ln(U_i)\right)^{1/k_1} \quad \}\text{if } U_j < w$$
$$D_i = \lambda_2 \left(-\ln(U_i)\right)^{1/k_2} \quad \}\text{otherwise} \qquad \ldots(6)$$

2. Further Results

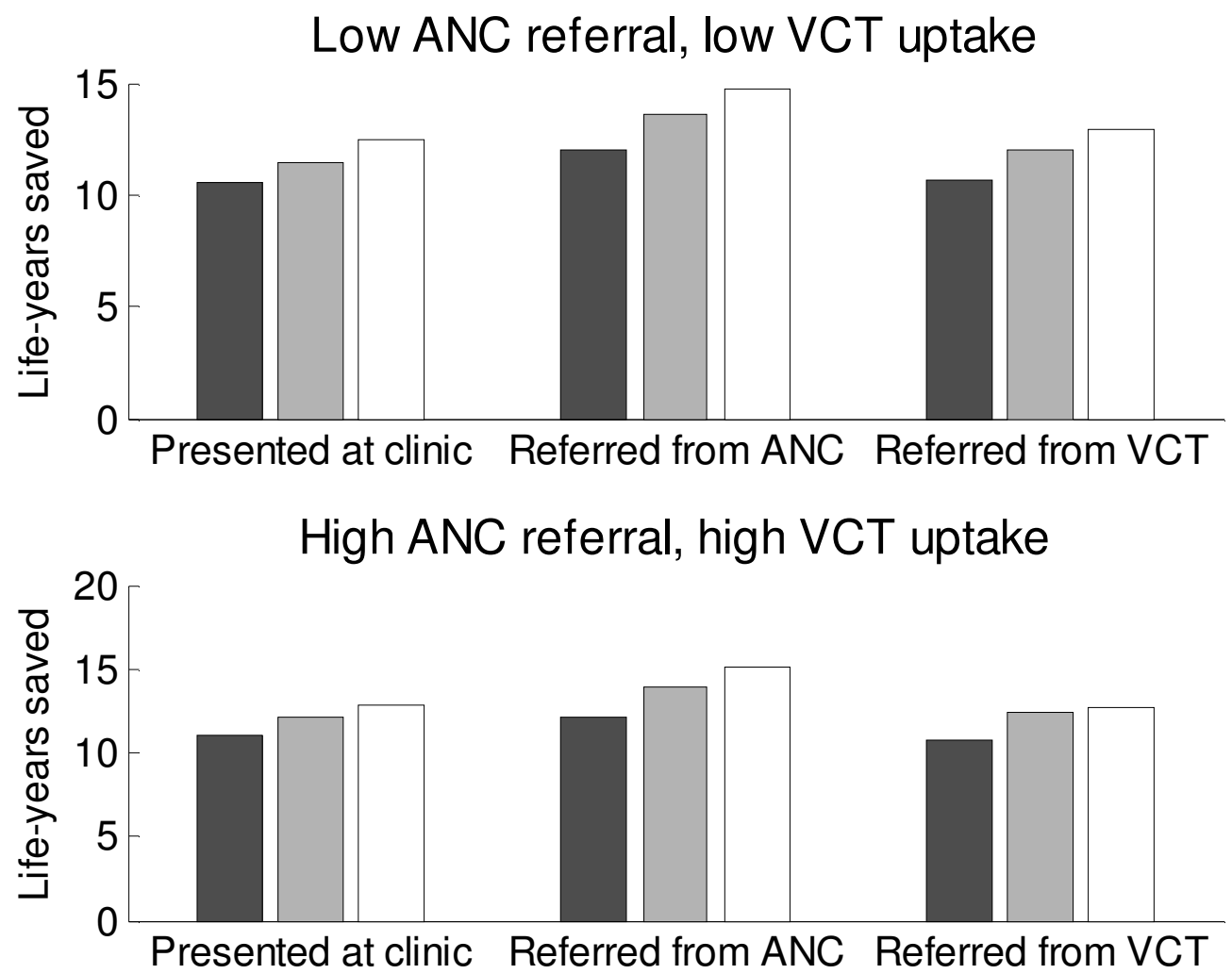

Figure A: What is the benefit of more frequent monitoring and how does this vary according to the ART initiation strategy and the route through which individuals are diagnosed?
The impact of increasing the frequency of monitoring individuals in care on average life-years saved per person diagnosed, by the route through which they enter care. Individuals in care are monitored every 12 months (dark grey bars), every 6 months (light grey bars) or every 3 months (white bars). In the upper panel, it is assumed that referral from ANC is low and uptake of VCT is low; in the lower panel, it is assumed that referral from ANC is high and uptake of VCT is high. ART is initiated with CD4 counts in the manner recommended by WHO (code 7; Table 2 in the main text). A 5% yearly drop-out from follow-up is assumed.

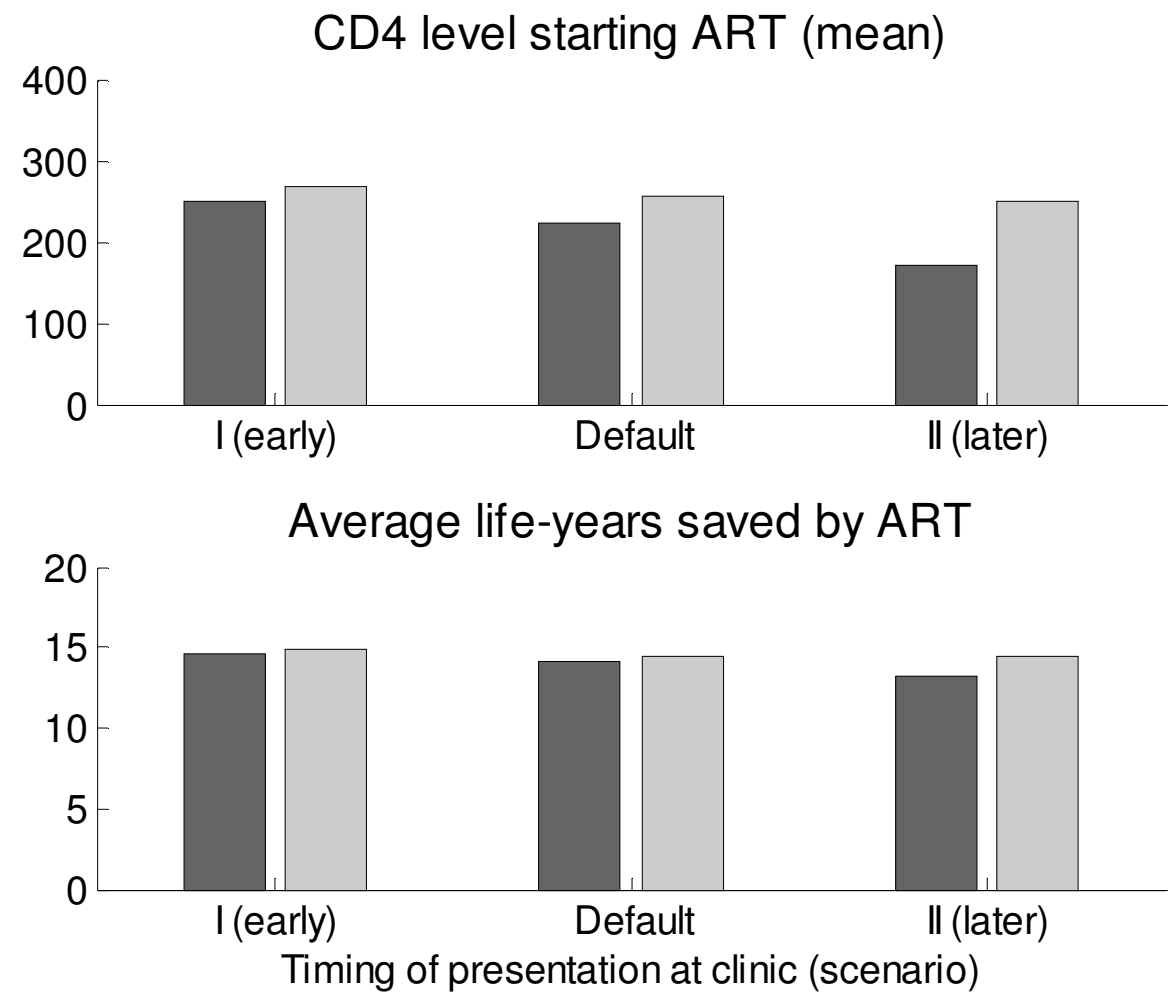

Figure B: How is the timing of individuals entering care associated with the timing of ART initiation and ART survival outcomes?

Alternative cohorts are compared where either all individuals enter early care through referral (from ANC or VCT; light grey bars) or individuals can only enter care late by presenting with symptoms (dark grey bars). Different assumptions are made about when individuals present at clinic (Figure 4): the 'default' scenario is based on data[27], the 'early' scenario represents individuals presenting at clinic is response to minor symptoms and the 'later' scenario represents individuals presenting at clinic after a longer delay. The measured outcomes are the mean CD4 count at which ART is begun (top panel) and the mean number of life-years saved per person that is treated (lower panel). Treatment is initiated using CD4 counts in the manner recommended by WHO (code 7; Table 2 in the main text). Individuals in care are monitored every six months and a 5% yearly drop-out from follow-up is assumed.

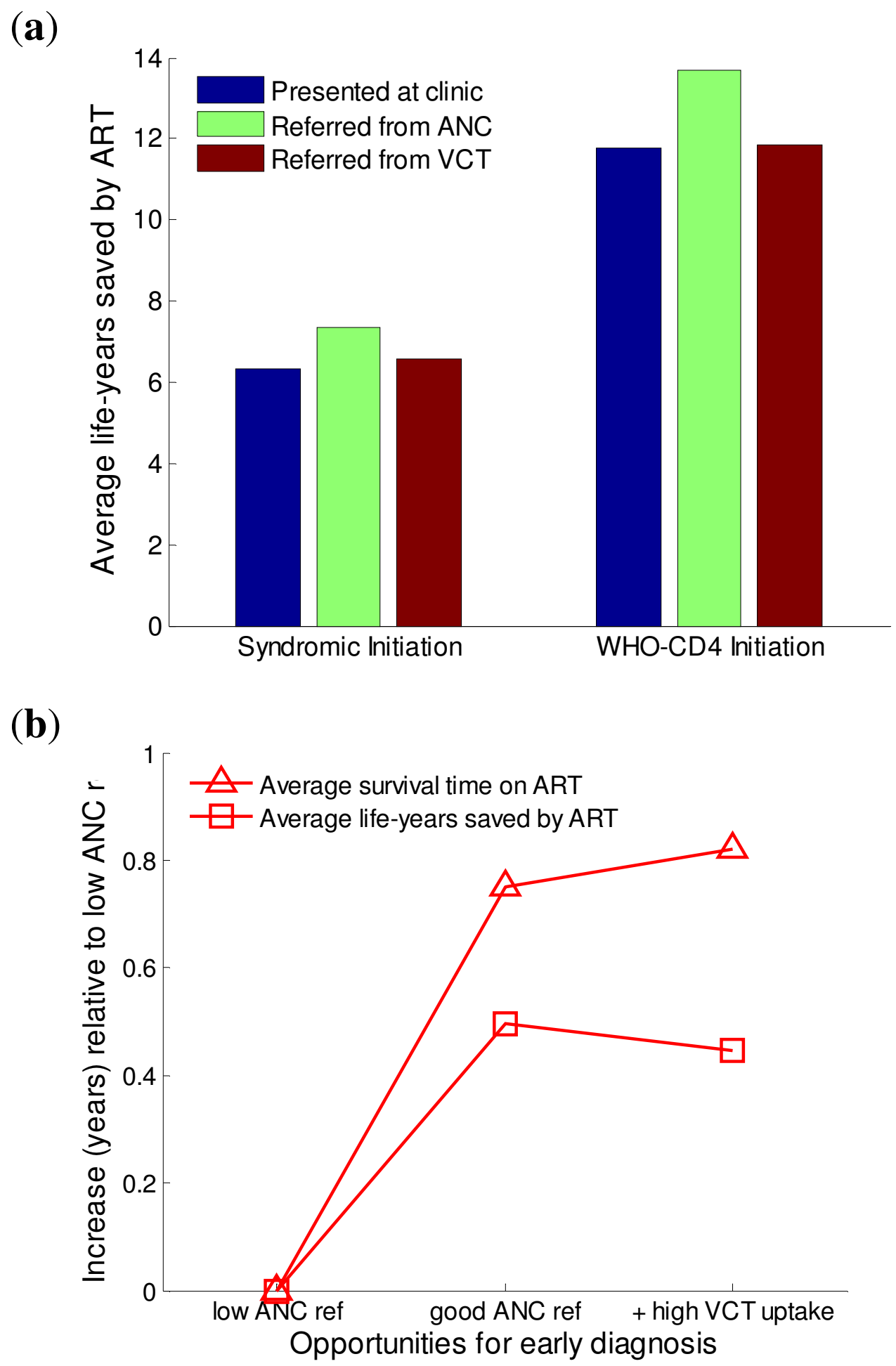

<u>Figure C: What are the individual level and population level effects of more individuals entering care through ANC and VCT referral?</u>

(**a**) Individual level. Average life-years saved by ART for those who enter the ART programme through ANC referral (blue bars), VCT referral (green bars) and those who present at clinic with symptoms (red bars), when ART is initiated syndromically or using CD4 counts in the manner recommended by WHO (codes 1 and 7; Table 2 in the main text). (**b**) Population level. The effect of increasing opportunities for diagnosis on the average survival time on ART (triangles) and average life-years saved by ART (squares). Values shown are the difference (in years) relative to where referral from ANC is low and VCT uptake is low. The initiation rule is the WHO recommendations (code 7). In both panels, individuals are monitored every 6 months; 5% yearly drop-out from follow-up is assumed; ANC referral is assumed to be high and VCT uptake is assumed to be low.

Increase in life-expectancy at infection (years)

		VCT uptake							
		5%	10%	20%	30%	40%	50%	60%	70%
		Syndromic Initiation							
ANC referral	**10%**	3.3	3.4	3.6	3.8	4.2	4.4	4.6	4.9
	**20%**	3.3	3.5	3.8	4.1	4.2	4.4	4.7	4.9
	**30%**	3.5	3.7	3.8	4.1	4.4	4.5	4.8	5.0
	**40%**	3.5	3.7	4.0	4.3	4.4	4.8	4.9	5.1
	**50%**	3.7	3.9	4.0	4.4	4.5	4.8	4.9	5.2
	**60%**	3.9	4.1	4.2	4.4	4.6	4.8	5.0	5.3
	**70%**	3.9	4.0	4.2	4.4	4.8	4.9	5.1	5.3
	**80%**	4.1	4.2	4.4	4.7	4.7	4.9	5.1	5.3
	**90%**	4.1	4.2	4.4	4.5	4.8	5.0	5.1	5.3
		WHO-CD4 Initiation							
	**10%**	6.7	6.8	7.5	8.3	8.4	9.1	9.6	10.1
	**20%**	7.1	7.3	7.7	8.4	8.8	9.3	9.9	10.2
	**30%**	7.3	7.4	8.0	8.5	8.9	9.4	9.9	10.3
	**40%**	7.6	7.8	8.4	8.7	9.3	9.7	10.2	10.4
	**50%**	7.9	8.0	8.4	8.9	9.4	9.6	10.0	10.6
	**60%**	7.9	8.2	8.7	8.9	9.5	9.9	10.4	10.7
	**70%**	8.3	8.4	8.8	9.1	9.6	10.0	10.4	10.8
	**80%**	8.6	8.6	8.9	9.3	9.8	10.1	10.5	11.0
	**90%**	8.6	8.7	8.9	9.6	10.0	10.2	10.6	11.0

Table A: What are the expected impacts of ART programme with intermediate levels of VCT uptake and ANC referral?

Improvements in life-expectancy at infection due to the availability of ART. This table is similar to Figure 2 in the main text but shows model output for intermediate values of ANC referral and VCT uptake. Patients are monitored every six months and there is no drop-out from follow-up.

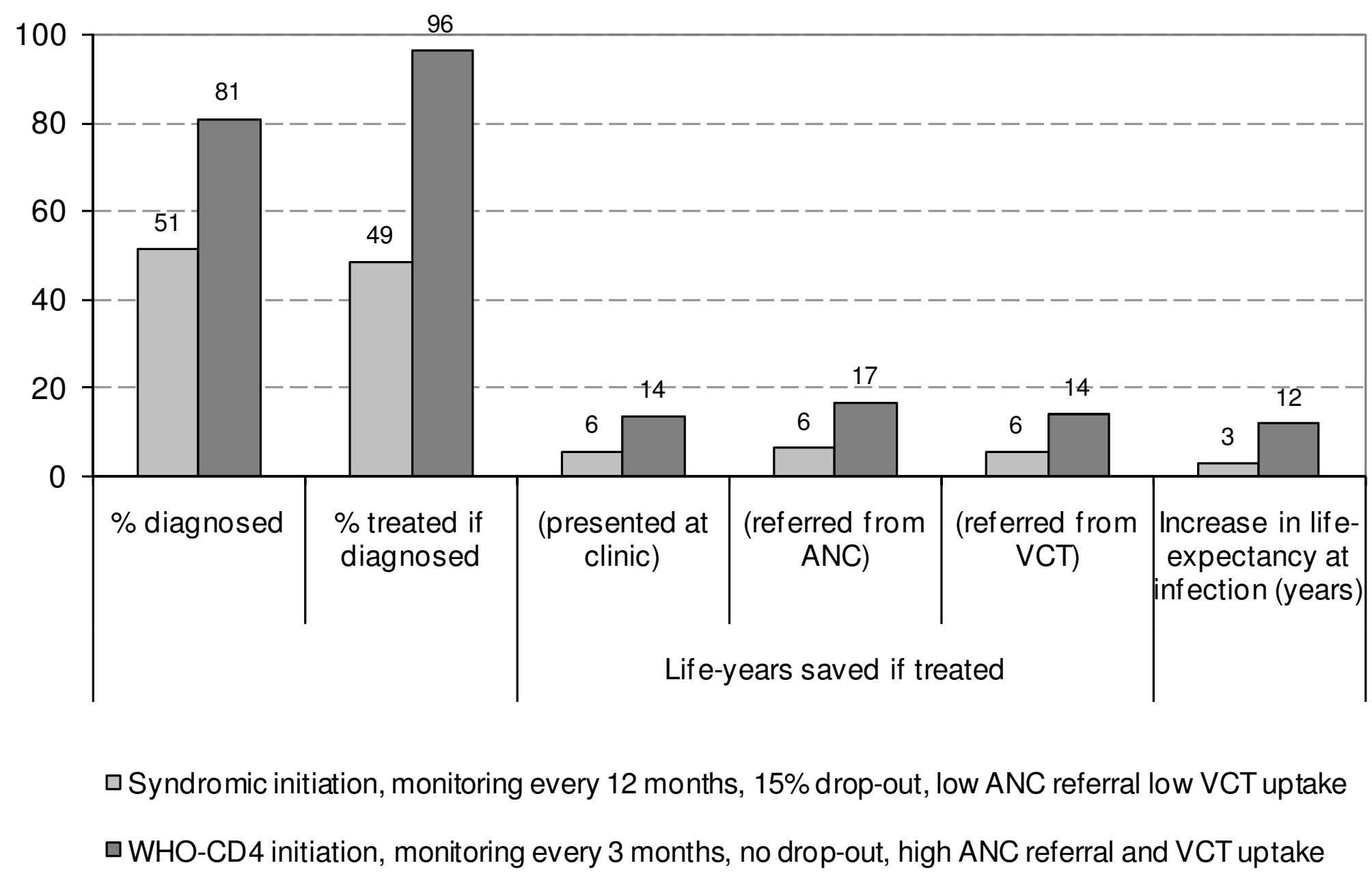

<u>Figure D: How does the ‘best-case’ ART delivery scenario lead to increased life-expectancy at infection?</u>

“Best” (dark grey bars) and “worst” (light grey bars) alternative ART programmes are compared. These scenarios are similar to the lowest and highest bars in Figure 2 in the main text, but here the components of the reported increase in life-expectancy at infection are quantified. With CD4 initiation, more frequent monitoring from earlier and less drop-out from follow-up, more individuals can enter care, more individuals in care can start ART, and individual on ART can have better survival chances.

Years saved per person diagnosed

Initiation Strategy	I<br>12	II<br>6 [II]	III<br>3 [III]	IV<br>12-3 (depn CD4) [IV]	V<br>6-24 (depn CD4 & age) [V]	VI<br>6-12 (depn age) [VI]	VII<br>On symptoms [VII]
	Interval between appointments (months)						
1	6.2	6.6	6.9	6.4	5.8	6.3	2.9
2	8.8	9.4	9.5	9.1	8.6	9.0	6.0
3	6.1	6.4	6.6	6.1	5.6	6.1	2.6
4	11.3	12.8	13.7	13.5	12.5	11.5	5.2
5	14.7	15.2	15.8	14.9	14.8	14.6	8.5
6	9.1	11.2	12.6	12.7	11.4	9.6	3.2
7	11.2	12.6	13.5	13.3	12.3	11.3	4.7
8	11.0	12.1	13.2	13.0	12.2	11.1	4.6
9	12.3	13.4	14.1	13.7	13.4	12.4	5.4
10	11.7	13.0	13.7	13.5	12.8	11.8	5.0

Years spent on ART (1000s)

Initiation Strategy	12	6 [II]	3 [III]	12-3 (depn CD4) [IV]	6-24 (depn CD4 & age) [V]	6-12 (depn age) [VI]	On symptoms [VII]
1	4.745	5.141	5.320	4.907	4.388	4.869	2.158
2	6.665	7.050	7.227	6.871	6.287	6.698	4.304
3	4.619	4.927	5.082	4.600	4.159	4.628	1.904
4	8.230	9.313	10.005	9.921	9.137	8.353	3.654
5	10.941	11.511	11.899	11.141	11.078	10.844	6.140
6	6.314	7.770	8.858	8.898	7.950	6.657	2.220
7	8.059	9.195	9.824	9.627	8.894	8.230	3.318
8	8.001	8.823	9.581	9.371	8.707	7.952	3.268
9	8.905	9.810	10.296	10.046	9.606	8.923	3.803
10	8.434	9.358	9.886	9.921	9.283	8.462	3.477

Years saved per year on ART

Initiation Strategy	12	6 [II]	3 [III]	12-3 (depn CD4) [IV]	6-24 (depn CD4 & age) [V]	6-12 (depn age) [VI]	On symptoms [VII]
1	0.8	0.8	0.8	0.8	0.8	0.8	0.8
2	0.8	0.8	0.8	0.8	0.8	0.8	0.9
3	0.8	0.8	0.8	0.8	0.8	0.8	0.9
4	0.9	0.9	0.9	0.9	0.9	0.9	0.9
5	0.8	0.8	0.8	0.8	0.8	0.8	0.9
6	0.9	0.9	0.9	0.9	0.9	0.9	0.9
7	0.9	0.9	0.9	0.9	0.9	0.9	0.9
8	0.9	0.9	0.9	0.9	0.9	0.9	0.9
9	0.9	0.9	0.9	0.9	0.9	0.9	0.9
10	0.9	0.9	0.9	0.9	0.9	0.9	0.9

Number of appointments (1000s)

Initiation Strategy	12	6 [II]	3 [III]	12-3 (depn CD4) [IV]	6-24 (depn CD4 & age) [V]	6-12 (depn age) [VI]	On symptoms [VII]
1	3.2	5.7	10.7	3.3	2.2	3.4	1.0
2	3.1	5.2	9.8	3.4	2.2	3.2	0.9
3	3.4	5.9	10.8	3.3	2.2	3.7	1.0
4	3.0	5.1	9.0	4.6	3.0	3.2	0.9
5	2.5	4.1	6.7	2.9	2.1	2.6	0.9
6	3.5	6.0	11.1	6.0	3.8	3.8	1.0
7	3.1	5.2	9.3	4.7	3.0	3.3	0.9
8	6.4	10.7	19.2	9.8	6.2	6.7	1.9
9	6.1	10.1	17.7	9.4	6.0	6.4	1.9
10	3.2	5.3	9.2	4.8	3.1	3.4	1.0

Number of CD4 tests per person diagnosed

Initiation Strategy	12	6 [II]	3 [III]	12-3 (depn CD4) [IV]	6-24 (depn CD4 & age) [V]	6-12 (depn age) [VI]	On symptoms [VII]
1	0.0	0.0	0.0	0.0	0.0	0.0	0.0
2	1.0	1.0	1.0	1.0	1.0	1.0	1.0
3	0.6	0.8	0.9	0.7	0.5	0.7	0.3
4	4.4	7.7	14.1	6.9	4.3	4.7	1.3
5	4.1	6.5	10.8	4.7	3.3	4.2	1.5
6	5.6	9.7	17.6	9.6	6.1	6.1	1.5
7	5.0	8.4	14.9	7.5	4.8	5.3	1.5
8	10.1	17.0	30.8	15.7	10.1	10.8	3.0
9	9.8	16.2	28.5	15.1	9.7	10.3	3.0
10	5.2	8.6	14.9	7.6	5.0	5.5	1.5

Table B: What is the expected impact of the alternative patient monitoring and ART initiation strategies?

Key indicator outcomes for alternative initiation rules and intervals between scheduled appointments. In all cases, the values relate to a cohort of 1000 HIV-infected individuals. ANC referral is assumed to be high, VCT uptake is assumed to be low and there is a 5% yearly drop-out rate.

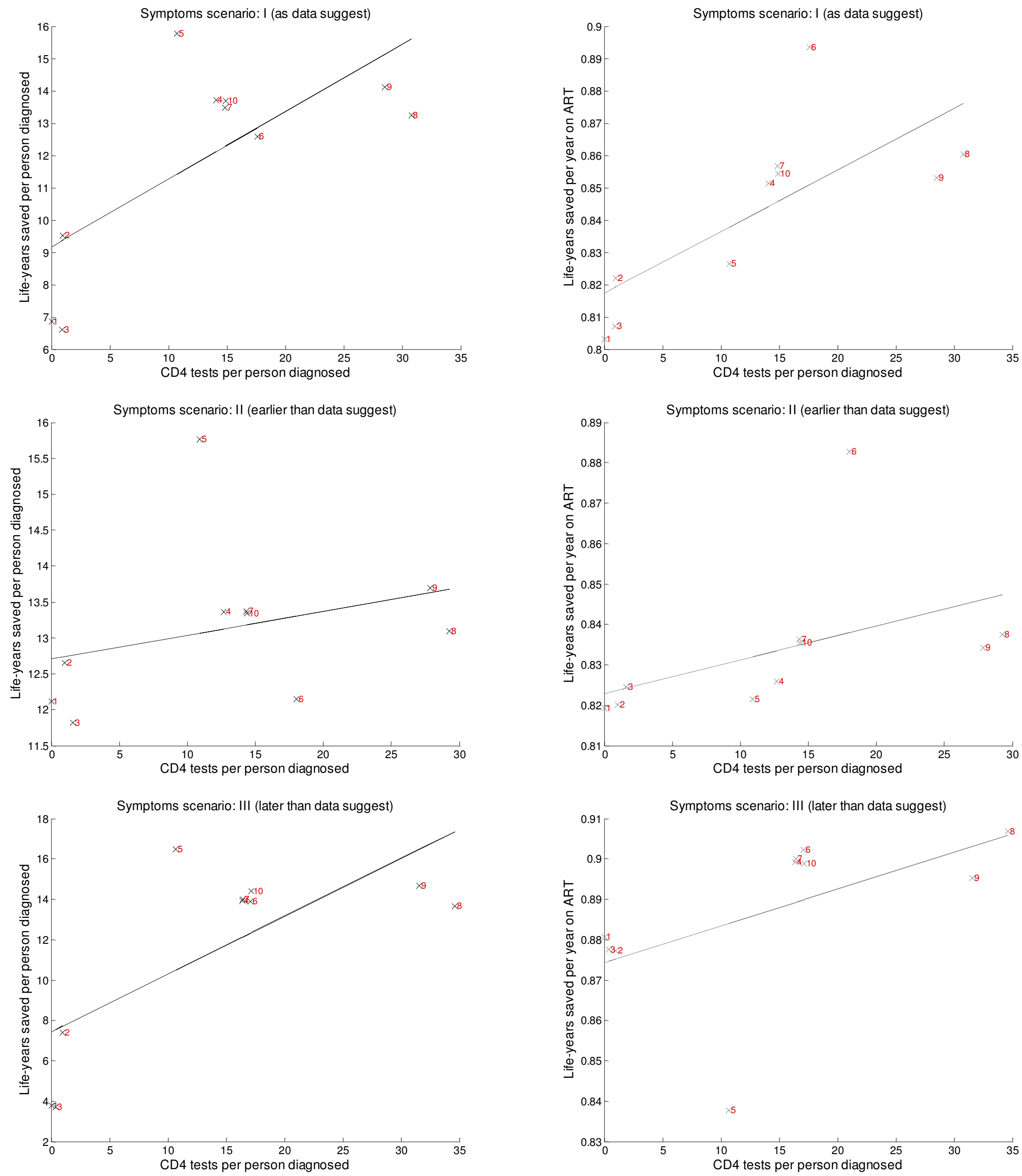

<u>Figure E: What is the overall relationship between the number of CD4 tests, life-years saved, and life-years saved per year on ART, and how does this vary according to the timing of symptoms?</u>
Comparison of possible initiation strategies in years saved per person diagnosed and years saved per year on ART plotted against the number of CD4 test used per person diagnosed. Lines are linear trends for ease of reading. The panels show the result when different assumptions on the timing of symptoms are made (see Figure 2). Points are labelled with the initiation code (Table 2 in the main text) to which they refer. Individuals are monitored for ART need every six months and there is a 5% yearly drop-out rate. Note that axis scales are different in each plot.

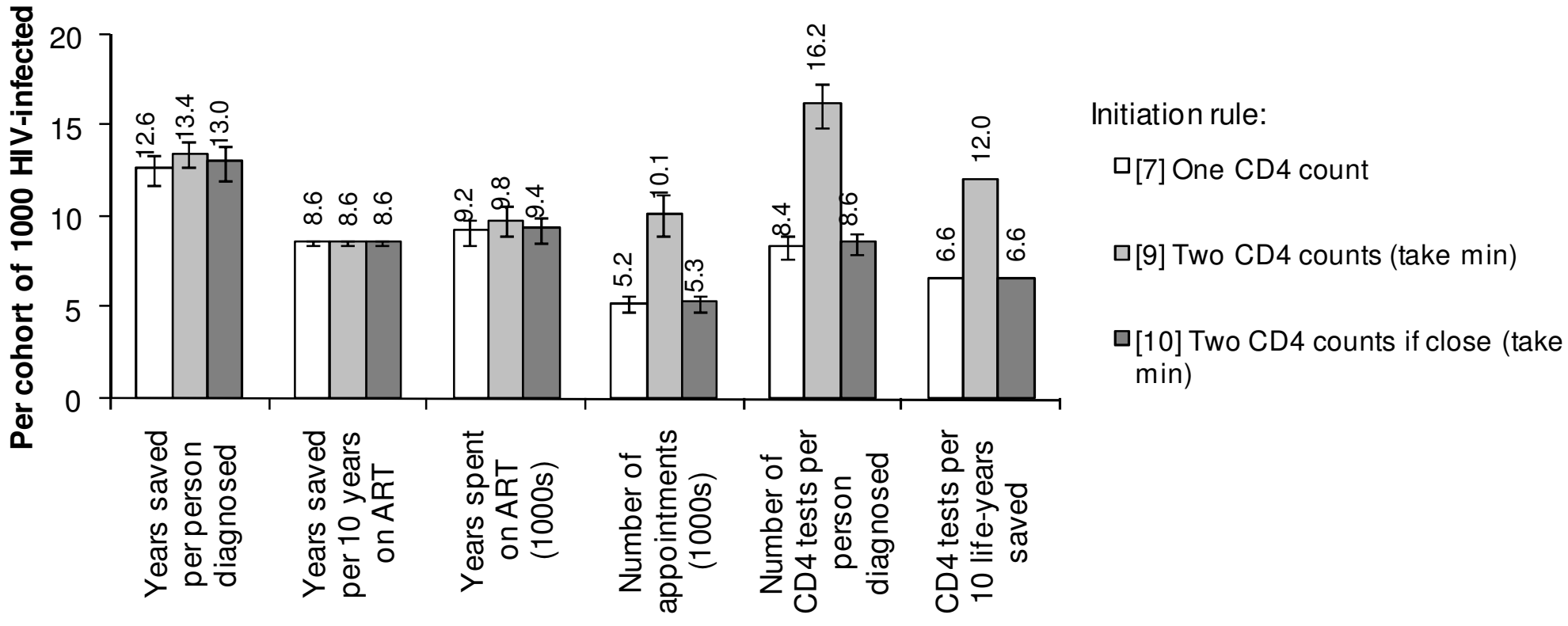

<u>Figure F: Is there an advantage in basing the decision to initiate ART on more than one CD4 cell count?</u>

Key indicator outcomes for alternative initiation rules. Rule 7 is initiation as per WHO recommendation using one CD4 count (code 7; Table 2 in the main text); rule 9 is the same but uses the minimum of two CD4 counts; and, rule 10 instead only uses a second CD4 count if the first is close to the appropriate threshold (see Table 2 in the main text for details). Appointments are scheduled for every 6 months, ANC referral is assumed to be high and VCT uptake is assumed to be low and there is a 5% yearly drop-out rate.

(a)

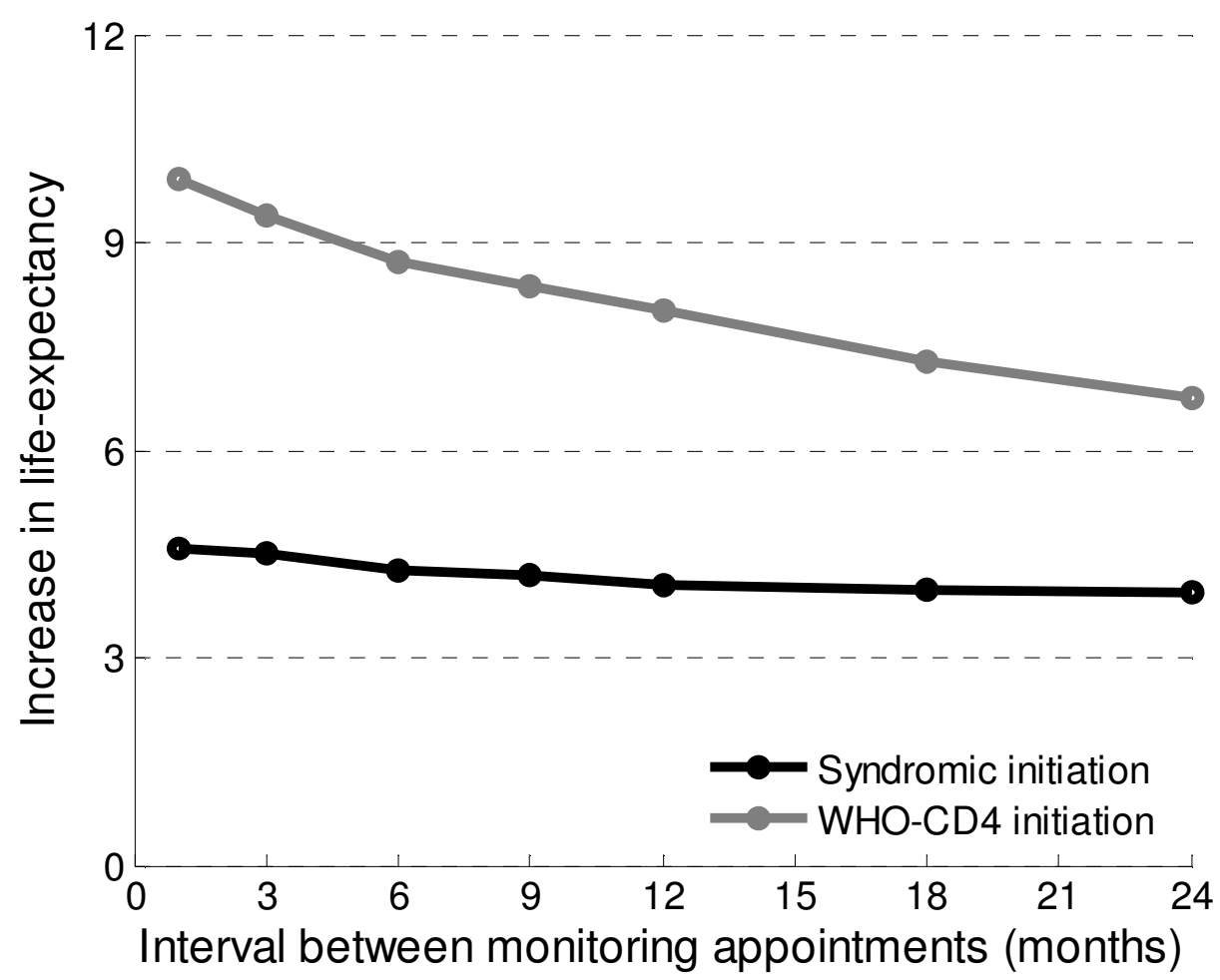

(b)

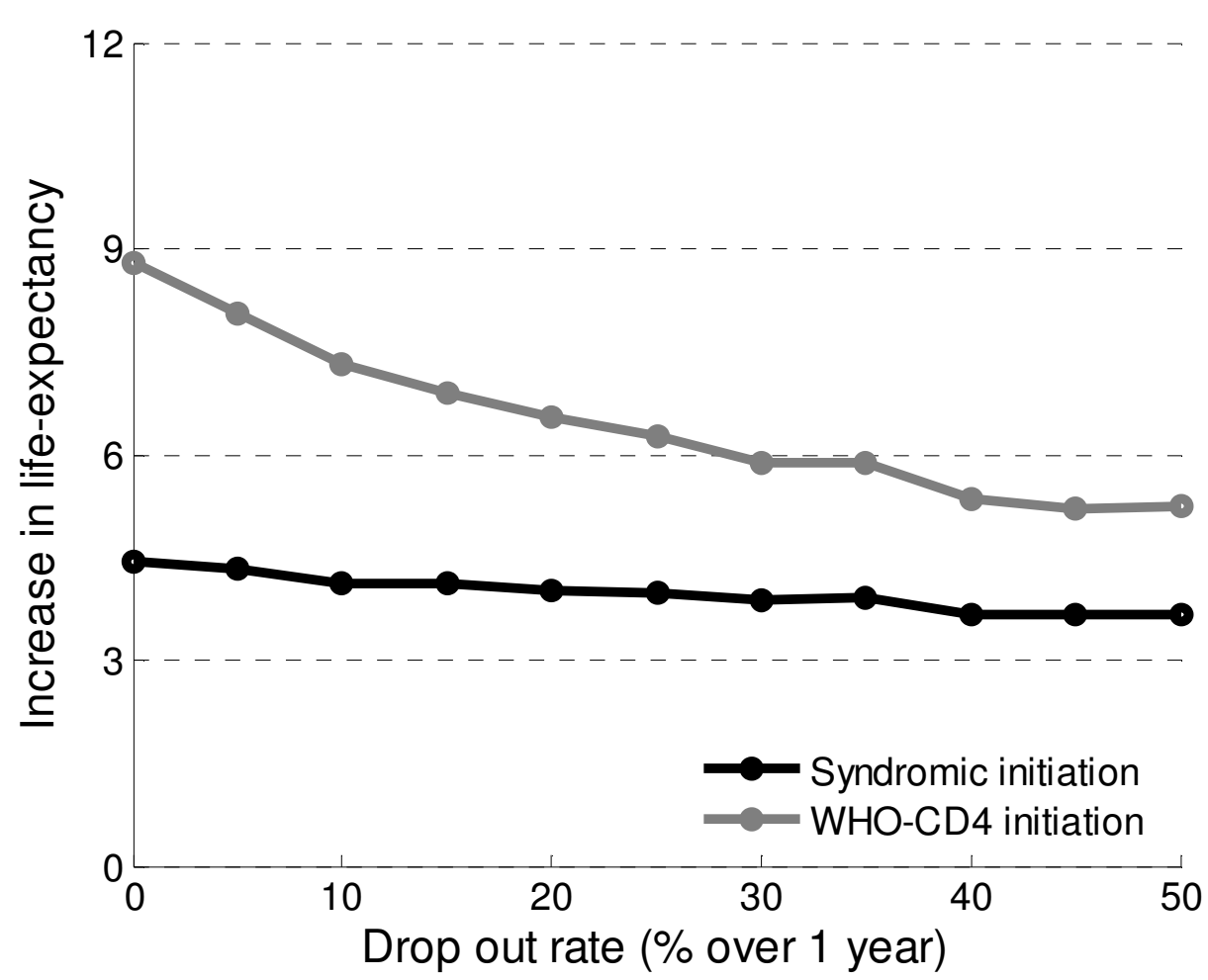

Figure G: How is the impact of ART programmes related to the intensity of patient monitoring (both frequency of scheduled appointments and the drop-out rate)?

Increase in life-expectancy at infection for ART programmes that initiate patients syndromically (black lines) or with CD4 cell count (grey lines). (**a**) Patients are scheduled to be monitored for the need for ART between every 24 months year and every month with no drop-out. (**b**) Patients are scheduled to be monitored every six months, but a fraction, between 0 and 50%, drop-out (i.e. do not attend the next appointment) every year. The rate of ANC referral is assumed to be 44% and VCT uptake is assumed to be 33%.

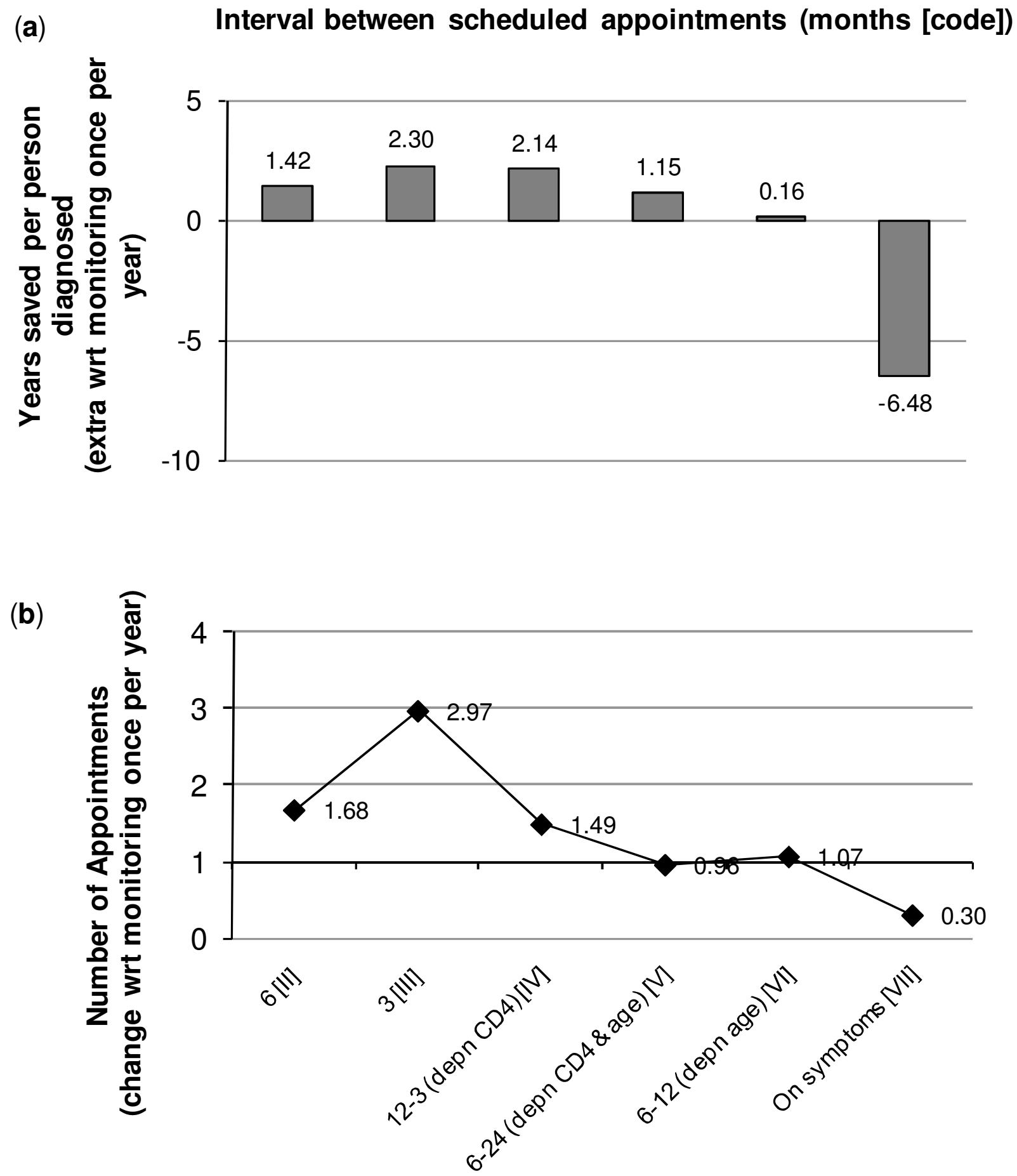

Figure H: What is the difference in impact of ART programmes that use the alternative patient monitoring strategies?

Comparison of different monitoring strategies (details in Table 1 in the main text) as (**a**) years saved per person (bars and numbers), and (**b**) total number of appointments (line), each given relative to scheduling appointment every 12 months. The initiation scenario is the WHO recommendation with CD4 counts (code 7, Table 2 in the main text), ANC referral is assumed to be high, VCT uptake is assumed to be low and there is 5% yearly drop-out from follow-up.

References

1. Gregson S, Garnett GP, Nyamukapa CA, Hallett TB, Lewis JJ, et al. (2006) HIV decline associated with behavior change in eastern Zimbabwe. Science 311: 664-666.
2. Dube S (In preparation) Should breastfeeding in the era of HIV/AIDS still be considered?
3. Lopman BA, Barnabas R, Hallett TB, Nyamukapa C, Mundandi C, et al. (2006) Assessing adult mortality in HIV-1-afflicted Zimbabwe (1998 -2003). Bull World Health Organ 84: 189-197.
4. Fraser C, Ferguson NM, de Wolf F, Anderson RM (2001) The role of antigenic stimulation and cytotoxic T cell activity in regulating the long-term immunopathogenesis of HIV: mechanisms and clinical implications. Proc Biol Sci 268: 2085-2095.
5. Margolick JB, Munoz A, Donnenberg AD, Park LP, Galai N, et al. (1995) Failure of T-cell homeostasis preceding AIDS in HIV-1 infection. The Multicenter AIDS Cohort Study. Nat Med 1: 674-680.
6. DeGruttola V, Lange N, Dafni U (1991) Modeling the progression of HIV infection. Journal of the American Statistical Association 86: 569-577.
7. McNeil AJ (1997) Bayes estimates for immunological progression rates in HIV disease. Stat Med 16: 2555-2572.
8. Lepri AC, Sabin CA, Pezzotti P, England PD, Phillips AN, et al. (1997) Is there a general tendency for CD4 lymphocyte decline to speed up during human immunodeficiency virus infection? Evidence from the Italian Seroconversion Study. J Infect Dis 175: 775-780.
9. Laurent C, Bourgeois A, Faye MA, Mougnutou R, Seydi M, et al. (2002) No difference in clinical progression between patients infected with the predominant human immunodeficiency virus type 1 circulating recombinant form (CRF) 02_AG strain and patients not infected with CRF02_AG, in Western and West-Central Africa: a four-year prospective multicenter study. J Infect Dis 186: 486-492.
10. Collaborative Group on AIDS Incubation and HIV Survival (2000) Time from HIV-1 seroconversion to AIDS and death before widespread use of highly-active antiretroviral therapy: a collaborative re-analysis. Collaborative Group on AIDS Incubation and HIV Survival including the CASCADE EU Concerted Action. Concerted Action on SeroConversion to AIDS and Death in Europe. Lancet 355: 1131-1137.
11. Morgan D, Mahe C, Mayanja B, Okongo JM, Lubega R, et al. (2002) HIV-1 infection in rural Africa: is there a difference in median time to AIDS and survival compared with that in industrialized countries? Aids 16: 597-603.
12. Manasa J, Musabaike H, Masimirembwa C, Burke E, Luthy R, et al. (2007) Evaluation of the Partec flow cytometer against the BD FACSCalibur system for monitoring immune responses of human immunodeficiency virus-infected patients in Zimbabwe. Clin Vaccine Immunol 14: 293-298.
13. Malone JL, Simms TE, Gray GC, Wagner KF, Burge JR, et al. (1990) Sources of variability in repeated T-helper lymphocyte counts from human immunodeficiency virus type 1-infected patients: total lymphocyte count fluctuations and diurnal cycle are important. J Acquir Immune Defic Syndr 3: 144-151.

14. Raboud JM, Haley L, Montaner JS, Murphy C, Januszewska M, et al. (1995) Quantification of the variation due to laboratory and physiologic sources in CD4 lymphocyte counts of clinically stable HIV-infected individuals. J Acquir Immune Defic Syndr Hum Retrovirol 10 Suppl 2: S67-73.
15. Levi FA, Canon C, Touitou Y, Reinberg A, Mathe G (1988) Seasonal modulation of the circadian time structure of circulating T and natural killer lymphocyte subsets from healthy subjects. J Clin Invest 81: 407-413.
16. Kagaayi J, Nakigozi G, Wawer M, Reynolds S. WHO Staging Criteria Versus CD4 Screening for Antiretroviral Eligibility in Rural Rakai District, Uganda; 2006 June, 2006; Durban, South Africa.
17. Kassa E, Rinke de Wit TF, Hailu E, Girma M, Messele T, et al. (1999) Evaluation of the World Health Organization staging system for HIV infection and disease in Ethiopia: association between clinical stages and laboratory markers. Aids 13: 381-389.
18. Schneider M, Zwahlen M, Egger M (2004) Natural history and mortality in HIV-positive individuals living in resource-poor settings. Available from wwwepidemorg.
19. Jaffar S, Grant AD, Whitworth J, Smith PG, Whittle H (2004) The natural history of HIV-1 and HIV-2 infections in adults in Africa: a literature review. Bull World Health Organ 82: 462-469.
20. Central Statistical Office [Zimbabwe] and Macro International Inc. (2000) Zimbabwe demographic and health survey 1999. Calverton, Maryland: Central Statistical Office and Macro International Inc.
21. Terceira N, Gregson S, Zaba B, Mason P (2003) The contribution of HIV to fertility decline in rural Zimbabwe, 1985-2000. Popul Stud (Camb) 57: 149-164.
22. Ross A, Van der Paal L, Lubega R, Mayanja BN, Shafer LA, et al. (2004) HIV-1 disease progression and fertility: the incidence of recognized pregnancy and pregnancy outcome in Uganda. Aids 18: 799-804.
23. Population Services International (2006) New Hope with New Start. Washington, D.C. available at http://www.psi.org/resources/pubs/New-Start-august-06.pdf p. (accessed December 2007).
24. Mulongo M, Wasagami F. High Rate of Discordance Among Clients Receiving Antiretroviral Therapy at TASO Uganda (abstract 118); 2006 June, 2006; Durban, South Africa.
25. De Cock KM, Marum E, Mbori-Ngacha D (2003) A serostatus-based approach to HIV/AIDS prevention and care in Africa. Lancet 362: 1847-1849.
26. Corbett EL, Dauya E, Matambo R, Cheung YB, Makamure B, et al. (2006) Uptake of workplace HIV counselling and testing: a cluster-randomised trial in Zimbabwe. PLoS Med 3: e238.
27. Adu-Sarkodie Y, Sangare A, d'Almeida OA, Kanmogne GD (1998) Distribution of CD4+ T-lymphocytes levels in patients with clinical symptoms of AIDS in three west African countries. J Clin Virol 11: 173-181.
28. Morgan D, Mahe C, Mayanja B, Whitworth JA (2002) Progression to symptomatic disease in people infected with HIV-1 in rural Uganda: prospective cohort study. Bmj 324: 193-196.
29. Morgan D, Ross A, Mayanja B, Malamba S, Whitworth J (1998) Early manifestations (pre-AIDS) of HIV-1 infection in Uganda. Aids 12: 591-596.
30. UNAIDS (2005) Evidence for HIV decline in Zimbabwe: a comprehensive review of the epidemiological data. www.epidem.org.

31. World Health Organization (2005) Interim WHO Clinical Staging of HIV/AIDS and HIV/AIDS Case Definitions for Surveillance: Africa Region. Geneva.
32. Egger M, May M, Chene G, Phillips AN, Ledergerber B, et al. (2002) Prognosis of HIV-1-infected patients starting highly active antiretroviral therapy: a collaborative analysis of prospective studies. Lancet 360: 119-129.
33. Braitstein P, Brinkhof MW, Dabis F, Schechter M, Boulle A, et al. (2006) Mortality of HIV-1-infected patients in the first year of antiretroviral therapy: comparison between low-income and high-income countries. Lancet 367: 817-824.
34. Egger M. Outcomes of Antiretroviral Treatment in Resource Limited and Industrialized Countries; 2007; Los Angeles.
35. De Cock KM, Fowler MG, Mercier E, de Vincenzi I, Saba J, et al. (2000) Prevention of mother-to-child HIV transmission in resource-poor countries: translating research into policy and practice. Jama 283: 1175-1182.
36. Stover J, Walker N, Grassly NC, Marston M (2006) Projecting the demographic impact of AIDS and the number of people in need of treatment: updates to the Spectrum projection package. Sex Transm Infect 82 Suppl 3: iii45-50.
37. Marston M, Zaba B, Salomon JA, Brahmbhatt H, Bagenda D (2005) Estimating the net effect of HIV on child mortality in African populations affected by generalized HIV epidemics. J Acquir Immune Defic Syndr 38: 219-227.
